# Spatiotemporal analysis of insecticide-treated net use for children under 5 in relation to socioeconomic gradients in Central and East Africa

**DOI:** 10.1186/s12936-020-03236-2

**Published:** 2020-04-22

**Authors:** Hana Kim, F. DeWolfe Miller, Andres Hernandez, Frank Tanser, Polycarp Mogeni, Diego F. Cuadros

**Affiliations:** 1 Department of Geography and Geographic Information Science, University of Cincinnati, Cincinnati, OH 45221 USA; 2 Health Geography and Disease Modeling Laboratory, University of Cincinnati, Cincinnati, USA; 3 Department of Tropical Medicine and Medical Microbiology and Pharmacology, University of Hawaii, Honolulu, HI USA; 4 Research Department of Infection & Population Health, University College London, London, UK; 5 Africa Health Research Institute, Durban, Kwazulu-Natal South Africa; 6 School of Nursing and Public Health, University of KwaZulu-Natal, Durban, Kwazulu-Natal South Africa

**Keywords:** Malaria, Insecticide-treated net, Disease mapping, Geospatial analysis, Central and East Africa

## Abstract

**Background:**

Insecticide-treated net (ITN) use is the core intervention among the strategies against malaria in sub-Saharan Africa (SSA) and the percentage of ITN ownership has increased from 47% in 2010 to 72% in 2017 across countries in SSA. Regardless of this massive expansion of ITN distribution, considerable gap between ownership and use of ITNs has been reported. Using data from more than 100,000 households in Central and East Africa (CEA) countries, the main aim of this study was to identify barriers associated with low ITN use and conduct geospatial analyses to estimate numbers and locations of vulnerable children living in areas with high malaria and low ITN use.

**Methods:**

Main sources of data for this study were the Demographic and Health Surveys and Malaria Indicator Surveys conducted in 11 countries in CEA. Logistic regression models for each country were built to assess the association between ITN ownership or ITN use and several socioeconomic and demographic variables. A density map of children under 5 living in areas at high-risk of malaria and low ITN use was generated to estimate the number of children who are living in these high malaria burden areas.

**Results:**

Results obtained suggest that factors such as the number of members in the household, total number of children in the household, education and place of residence can be key factors linked to the use of ITN for protecting children against malaria in CEA. Results from the spatiotemporal analyses found that although total rates of ownership and use of ITNs across CEA have increased up to 70% and 48%, respectively, a large proportion of children under 5 (19,780,678; 23% of total number of children) still lives in high-risk malaria areas with low use of ITNs.

**Conclusion:**

The results indicate that despite substantial progress in the distribution of ITNs in CEA, with about 70% of the households having an ITN, several socioeconomic factors have compromised the effectiveness of this control intervention against malaria, and only about 48% of the households protect their children under 5 with ITNs. Increasing the effective ITN use by targeting these factors and the areas where vulnerable children reside can be a core strategy meant to reducing malaria transmission.

## Background

Malaria is a vector-borne disease caused by the parasite *Plasmodium* and transmitted by the female *Anopheles* mosquito. It is the leading cause of death in sub-Saharan Africa (SSA) [1]. Children under 5 years old are the most vulnerable population, accounting for 61% of all deaths in children under 5 globally, and 70% in SSA [1]. Although the total number of new malaria cases has declined by an estimated 37% in SSA [2], the region is still suffering a significant burden of malaria morbidity, experiencing 92% (200 million cases) of the estimated 219 million cases of malaria worldwide [1]. In 2015, the World Health Organization (WHO) endorsed the Global Technical Strategy for Malaria 2016–2030 [3], with its global targets of reducing malaria incidence and mortality rates by 90% in 2030, with milestones for measuring progress in 2020 and 2025 [2, 3]. As a result of this initiative, countries like Rwanda reported in 2017 a reduction in new cases for the first time since 2011 (430,000 fewer cases compared with 2016) [1].

Insecticide-treated net (ITN) use remains as one of the core interventions among the strategies against malaria in SSA along with indoor residual spraying, and intermittent preventive treatment for pregnant women, and drugs and diagnostics [1]. ITN has contributed to a ~ 50% reduction in total malaria incidence and ~ 55% reduction in mortality rates in children under 5 in SSA [1, 2, 4]. The Roll Back Malaria Partnership sets a goal to scale up ITN coverage and use, targeting pregnant women and children under 5 [5]. With increased funding of US$ 3.1 billion from governments of malaria-endemic countries and international donors [1], 220 million ITNs have been distributed globally, with approximately 175 million ITNs (81% of total distributed ITNs) distributed across SSA [1]. The overall percentage of households with at least one ITN in SSA has increased from 47% in 2010 to 72% in 2017 [1].

Regardless of this massive expansion of ITN distribution, a considerable gap between ownership and use of ITNs has been reported. Nearly 40% of households in SSA do not use ITNs to protect their children while they sleep [1]. This minimizes the impact of ITNs on malaria-related morbidity and mortality in these vulnerable populations [6]. The factors associated with the lack of use of ITNs have not been well identified and described. The identification of the barriers that diminish the use of ITNs is critical for understanding the impact of the ITN intervention programmes, and ultimately for ensuring the success of the Global Technical Strategy for Malaria 2016–2030 goals.

Previous studies attempted to assess the socio-economic factors associated with ITNs use in several African countries found that factors that have been identified to increase the likelihood of ITNs use among households living with at least a child under 5 included: small-size households with equal or less than four household members [7–10], urban residents [11–13], living in improved housing [14], wealthier households, and households with short distance to retail stores [15]. Moreover, high level of education has been linked to appropriate ITNs use [10–13, 16–18]. For example, a study in Democratic Republic of Congo (DRC) pointed out that reasons for not using ITNs were associated with misconception about ITNs’ usage, and concluded that education can reduce these misconceptions [17]. Several critical issues remain to be addressed in order to identify the determinants affecting the use of ITNs in this African region. First, little attention has been paid to factors affecting ITN use in local areas characterized by low ITN use across the SSA region. Most studies that assessed the use of ITNs have been conducted on large geographical administrative units such as country and province, thereby obscuring important localized aspects of the ITN use [6, 19, 20]. Second, recent studies of spatial dynamics of ownership and use of ITNs in SSA only assessed the changes in the geographical distribution of ITNs [19–21]. These studies estimated changes in ownership and use of ITNs using geographical methodologies in limited African settings [20]. Moreover, accurate estimations of the density and location of the vulnerable population at high-risk of malaria living in high malaria burden areas but with low ITNs use have not been evaluated.

Against this background, using data collected from more than 100,000 households in 11 countries in Central and East Africa (CEA) of SSA region, the barriers associated with low ITN use in CEA were identified. Moreover, the analyses were strengthened by including geospatial estimates of the number and location of vulnerable children under 5 living in areas with high malaria and low ITN use.

## Methods

### Data sources

**Fig. 1:**
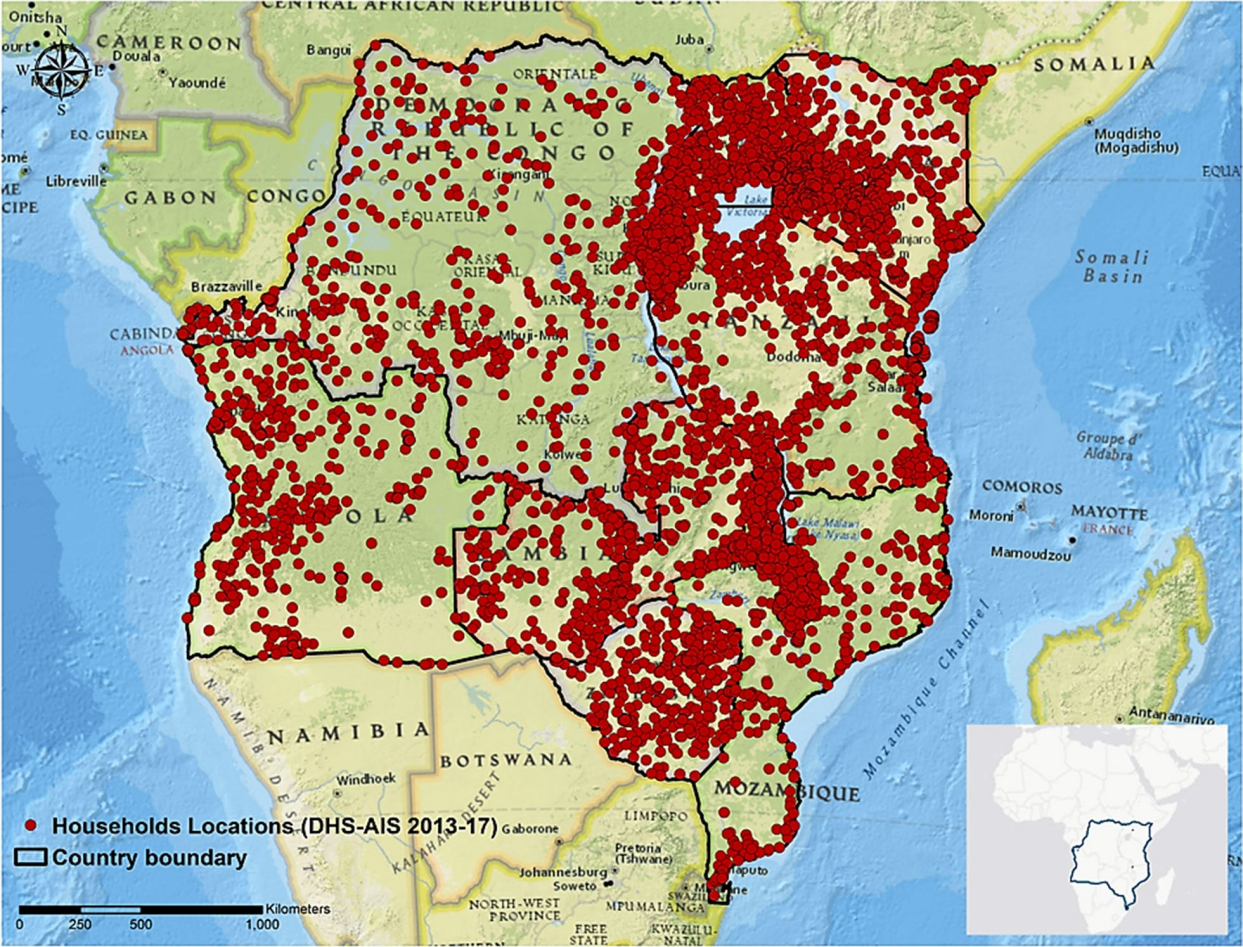
The overall study area (bottom right) and Demographic and Health Survey sample locations in DHS-MIS 2013–2017. Maps were created using ArcGIS^®^ by Esri version 10.5 (http://www.esri.com) [59], and basemaps were obtained from ESRI and National Geographic available at ArcGIS Online basemaps [60]

The main sources of data for this study were the Demographic and Health Surveys (DHS) and Malaria Indicator Surveys (MIS) conducted in 11 countries in CEA during the period between 2013 and 2017. Inclusion of these countries in the study was based on data availability related to ITN ownership and use as well as geographic position system information for each location sampled. Data from the following countries were included in the analyses: Angola [22], Burundi [23], DRC [24], Kenya [25], Malawi [26], Mozambique [27], Rwanda [28], Tanzania [29], Uganda [30], Zambia [31], and Zimbabwe [32]. The DHS and MIS are cross-sectional household surveys designed to collect nationally representative data on population, health, and socioeconomic parameters [33]. These surveys are conducted approximately every 5 years in each target country with large sample sizes (usually between 5000 and 30,000 households). The study designs included primary sample units (PSUs), selected using a two-stage sampling procedure proportional to the population of the entire country. The global positioning system was used to identify and record the geographical coordinates of each PSU’s central location (Fig. 1). A total of 191,521 households participated in the surveys, including 7315 PSUs (2452 in urban and 4863 in rural areas). Men and women aged 15–49 years in the selected households were eligible for the surveys conducted by DHS. Therefore, the population of this study was restricted to these age groups. The analyses were also restricted to households with children under 5 years of age, resulting to 107,500 households included in the final analyses.

Additionally, DHS and MIS survey data were obtained collected in the previous wave for each country (surveys conducted during 2007 to 2011) with a total of 4794 PSU locations (1418 in urban and 3376 in rural areas) to assess the spatiotemporal changes of ownership and use of ITNs across countries between the two time-periods (2007–2011 and 2013–2017).

### Outcome variables

Two outcome variables were analysed in the study: (1) *ownership of ITNs* and (2) *use of ITNs*. The binary, yes or no, answer to the question *“Does your household have any ITN?”* was used as the measure of ITN ownership. The categorical answer to the question *“Who slept under this mosquito net last night?”* was used as the measure of ITNs use among households that own ITNs. The authors reclassified the responses of the question, *no children, all children, and some children* into two categories: at least one child slept under a net in the household that answered *all children or some children* to the question, (a household that uses ITNs for protecting their children when they sleep) and a household in which no children slept under the ITNs, who answered *no children* to the question (a household in which ITNs is not used for children).

### Socioeconomic and malaria endemicity variables

Socioeconomic and demographic variables were obtained from the DHS and MIS dataset for each country. Eight socioeconomic variables were used: total number of household members, number of children under 5 in the household, place of residence, highest level of education the head of the household, housing qualities (main floor, wall, and roof materials of the house), and DHS wealth index. Since the analyses encompassed a considerable amount of data including several socioeconomic and demographic variables from 11 CEA countries, each variable was classified into binary form for precise interpretations of the results and to be able to perform comparisons between countries. All variables were converted either into binary variables using the DHS-MIS definitions (for categorical values) or the median value (for continuous variables). For example, DHS and MIS classify highest education level of the household member as *no education, primary*, *secondary, and higher*. In this study, the highest education level of the household member was classified as *high* if it was secondary or higher, and all other categories were classified as *low*. A complete definition and description of each socioeconomic variable included in the analysis can be found in Additional file 1.

Two factors relating to malaria endemicity and transmission, estimated *Plasmodium falciparum* parasite rate (*Pf*PR_2-10_) estimated for the year 2017 and average land travel friction per metre estimated for the year 2015, were obtained for each PSUs from the Malaria Atlas Project [34–36]. *Pf*PR_2–10_ was included as a malaria variable to assess the effect of exposure to malaria infection. The algorithm for the estimation of *Pf*PR_2–10_ has been described elsewhere [34]. Average land travel friction per metre was used as a transmission variable, representing allocated fastest speed of travel based on the types of travel mode estimated within the pixel, which is expressed in units of minutes required to travel one metre [35]. This measure represents not only the type of residence (urban or rural) but also the accessibility and mobility in these areas. Thus, it can be a potential socioeconomic indicator of accessibility and development besides other DHS variables that are not possible to measure and could have an impact on the health outcome of the population [35, 37]. PSUs were overlaid onto the two surfaces maps mentioned above to extract values of *Pf*PR_2–10_ and average land travel speed at each PSU location, which were further classified into binary variables and assigned to each household. Each median value for *Pf*PR_2–10_ and average land travel speed was calculated using extracted values and used to classify each value into binary value (high and low). All spatial analyses and maps were generated using ArcGIS version 10.5 [38]. A complete definition and further description of each variable included in the analysis can be found in Additional file 1.

### Statistical analysis

Robust logistic regression models for each country were built to assess the association between ITN ownership or ITN use among households that own ITNs and the variables previously described, adjusting for the PSU-level effect. First, a preliminary bivariate analysis was conducted between the outcome variables, ownership and use of ITNs, and each of the 10 covariates for each country. The significantly associated variables at *p *< *0.05* level with ownership of ITNs or use of ITNs were used to generate the final adjusted multivariable models using the backward elimination strategy. Household and PSU-level data were weighted and adjusted for two-stage cluster sample design according to recommendations of DHS [39]. Odds ratios (ORs) from each significant variable in each country were combined to determine a pooled OR across all country surveys using a random effects meta-analysis [40]. All statistical analyses were conducted using R statistical software v3.5.2 and SAS software v.9.4 [41, 42].

### Spatiotemporal dynamics of ITN ownership and use

Continuous surface maps of ITN ownership for both periods were generated to compare the spatial distribution of the net ownership in the two survey cycles 2007–2011 and 2013–2017. First, the prevalence of households that owned ITNs (*p*_*ON*_) was estimated at each PSU. The prevalence of ITN ownership at location *i* was defined as *p*_*ON*_*i* = *H*_*ONi*_*/HH*_*i*_, where *H*_*ONi*_ denotes the number of households that owned ITNs, and *HH*_*i*_ denotes the total number of households with at least one child under 5 at location *i*. Second, kriging interpolation method was used to produce continuous surface maps of the prevalence of ITN ownership [38, 43]. The method of ordinary kriging, which has been widely used in spatial mapping [43–46], was used to predict the values of variables (prevalence of ITN ownership) at unmeasured locations by estimating a variogram of weighted averages of the data [47]. Each map was generated in a raster format at 5 km x 5 km pixel resolution. Third, spatial changes in the ITN ownership between the first and second survey periods were estimated as the absolute difference between the two prevalence maps. Likewise, continuous surface maps of ITN use were created for both sampling periods. Prevalence of households that use ITNs at location *i* (*p*_*UN*_*i*) was defined as *p*_*UN*_*i* = *H*_*UNi*_*/HH*_*i*_, where *H*_*UNi*_ denotes the number of households with at least one child under 5 that used ITNs, and *HH*_*i*_ denotes the total number of households with at least one child under 5 at location *i*.

### Density map of children aged under 5 at high-risk of malaria

A density map of children under 5 living in areas at high-risk of malaria and low ITN use was generated to estimate the number of children who are living in areas with high *Pf*PR_2–10_, but low use of ITNs. First, the proportion of ITN use was reclassified into a binary variable, as areas with high ITNs use (> 60%), and areas with low ITN use (≤60%). Based on the results of ITN use by country, the average proportion of household using ITNs for their children under 5 was less than 50% (ranged from 13% to 65%), which is consistent with the percentage of population sleeping under an ITN in SSA [1]. Half of the countries had more than 50% of their household using ITNs for their children under 5, and only two countries (Rwanda and Uganda) had more than 60% of their household using ITNs for their children under 5. Thus, a 60% was set as the cutoff to classify areas with low and high ITN use. Second, to identify areas with high malaria transmission intensity, *Pf*PR_2–10_ was reclassified into a binary variable, with areas with high malaria transmission intensity (*Pf*PR_2–10_ > 0.1) and areas with low malaria transmission intensity (*Pf*PR_2–10_ ≤ 0.1). Third, the high-risk areas of malaria transmission were defined by combining these two maps of low ITNs use and high *Pf*PR_2–10_. Lastly, a 5 km x 5 km pixel resolution map of the density of children under 5 living in the high *Pf*PR_2–10_ area was generated by combining the high-risk areas of malaria transmission map with the children under 5 population density maps. Population density throughout the countries included in the study was obtained from the WorldPop database [48, 49]. This database was used to extract estimates of the 2015 population density in the different countries. Further methodological details used by WorldPop are described elsewhere [48, 49]. Estimates of population densities from the WorldPop dataset were obtained as a raster image with a resolution of 100 m x 100 m pixel. For consistency with the resolution of the maps generated previously, the resolution of the raster file was reduced to 5 km x 5 km pixel. Population density map for children under 5 in the study area was generated using estimates from the Demographic Profile of African Countries report [50].

## Results

### General description and malaria prevalence

Of the 107,500 households with at least one or more children under 5 across the study area, 56,874 households had only one child under 5 (53%). The head of the household had low education in 71,809 households (67%), and 48,440 of the households had low-income (45%). Less than half of the households had a house built with unfinished wall and roof material (49% and 41%, respectively), whereas 66% of households had a house built with unfinished floor material. Further household descriptive statistics can be found in Additional file 1: Table S1. Overall malaria prevalence in children under 5 across countries was 20%, ranging from 2% (Rwanda) to 40% (Mozambique). Rwanda, Kenya, and Tanzania showed lower malaria prevalence (2%, 5%, and 7%, respectively).

### National-level changes in the ownership and effective use of ITNs

**Fig. 2:**
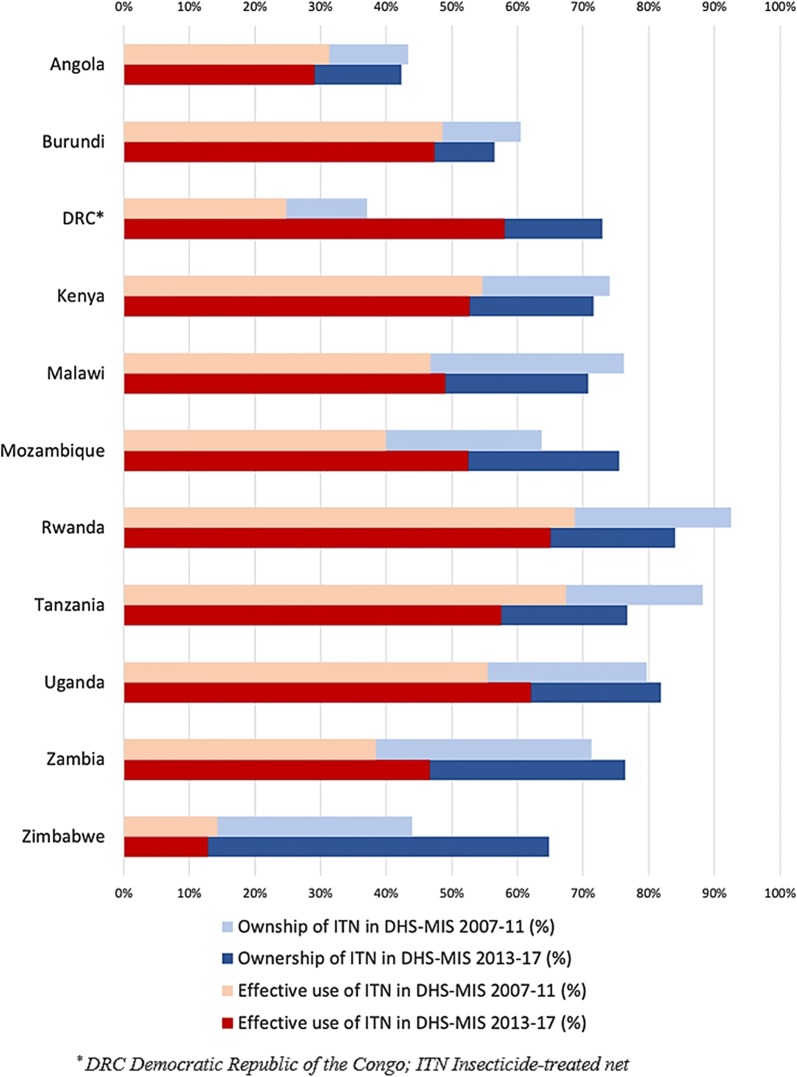
National-level changes in the ownership of ITNs (Blue) and effective use of ITNs (Red) between DHS-MIS 2007–2011 (lighter) and DHS-MIS 2013–2017 (darker) (alphabetical order)

The average proportion of ITN ownership among households living with at least one child under 5 in the 11 countries increased by 4% between the survey years, from 66% (ranging from 37% in DRC to 93% in Rwanda) in DHS-MIS 2007–2011 to 70% (ranging from 42% in Angola to 84% in Rwanda) in DHS-MIS 2013–2017. Similarly, the average proportion of households that used ITNs for their children under 5 in the total sample increased by 4%, from 44% (ranging from 14% in Zimbabwe to 69% in Rwanda) in DHS-MIS 2007–2011 to 48% (ranging from 13% in Zimbabwe to 65% in Rwanda) in DHS-MIS 2013–2017 (Fig. 2).

The country with the highest improvement in both ownership and use of ITN was DRC, with a 36% increase in ITN ownership (from 37% in 2007 to 73% in 2013–2014) and 33% increase in ITN use (from 25% in 2007 to 58% in 2013–2014). By contrast, the largest decrease in both ownership and use of ITNs was observed in Tanzania, with a 12% reduction (from 88% in 2010 to 77% in 2015–2016) and 10% (from 67% in 2010 to 57% in 2015–2016) decrease, respectively (Fig. 2). Malawi and Zimbabwe reported an opposite change in the patterns of both ownership and ITN use. In Malawi, ITN ownership decreased by 5%, whereas ITN use increased by 2%. In contrast, Zimbabwe showed an increase of 21% in the ownership of ITNs, while ITNs use decreased by 1% during the two survey waves (Fig. 2).

### Socioeconomic and malaria endemicity variables

**Fig. 3:**
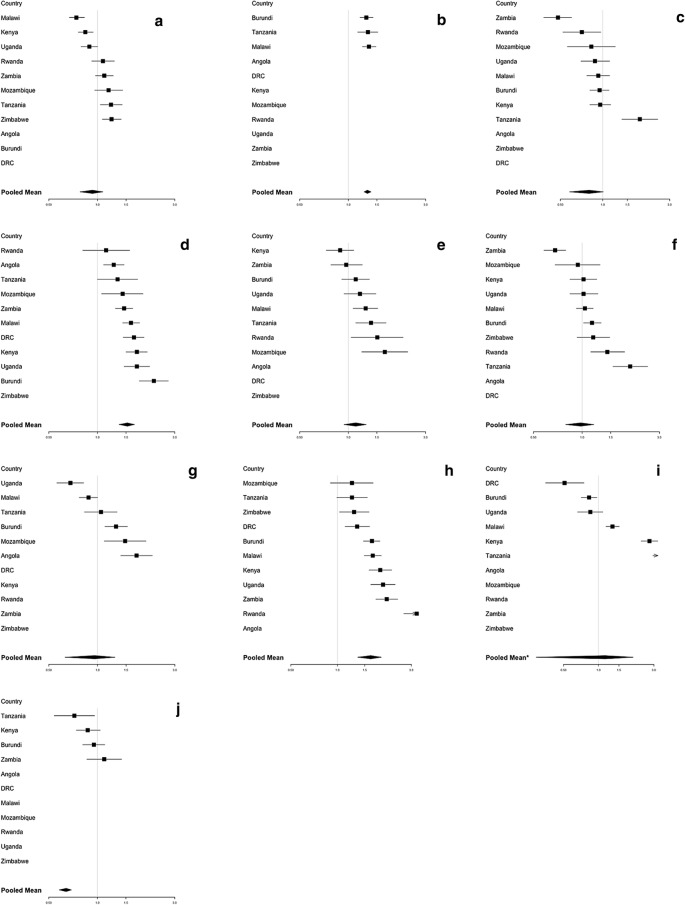
Association between ownership of ITNs and statistically significant covariate factors. **a** Households with a large number of members (more than 4 people); **b** Households with more than one child under 5; **c** Households located in urban areas; **d** Households with high education level; **e** Houses built with a finished floor material; **f** Houses built with a finished wall material; **g** Houses built with a finished roof material; **h** Wealthy households; **i** Households located in high malaria parasite rate areas (≥ 0.09); **j** Households located in high average land travel per metre areas (≥ 0.003). ^1^*DRC* Democratic Republic of the Congo; *ITN* Insecticide-treated net. ^2^The solid line represents the null value (no difference between groups). ^3^Odds ratios (OR) are adjusted for variables which are significant in unadjusted analyses. ^4^Error bars show 95% confidence intervals

Table 1 shows the statistically significant socioeconomic and malaria endemicity variables associated with the ownership of ITNs (Table 1a) and the use of ITNs (Table 1b) in bivariate regression analysis per country. Households with more than one child under 5 have higher odds of having ITNs in the household (OR = 1.31; 95% confidence interval [CI] 1.25–1.38). Likewise, wealthy households and households with high education showed higher odds of having ITNs in the household (OR = 1.64; 95% CI 1.35–1.92 and OR = 1.53; 95% CI 1.36–1.69, respectively). On the contrary, average land travel friction per metre showed non statistically significant lower odds of having ITNs in the households (OR = 0.90; 95% CI 0.71–1.09) (Fig. 3).

**Table 1:**
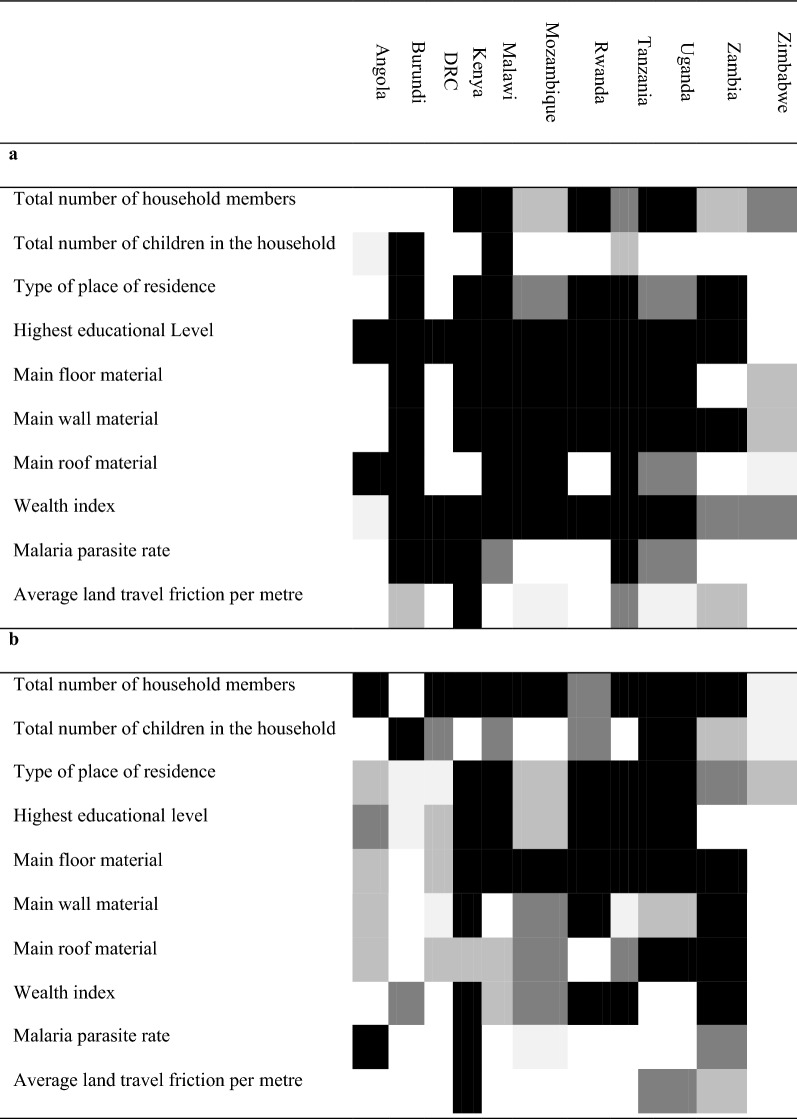
Repression of significant factors associated with the ownership of ITNs (**a**) and the use of ITNs (**b**) identified from the bivariate regression models^1, 2^

^1^Darker colour shading equivalent to higher statistical significance for the covariate indicated in the row, for the country in the corresponding column

^2^*DRC* Democratic Republic of the Congo, *ITN* Insecticide-treated net

**Fig. 4:**
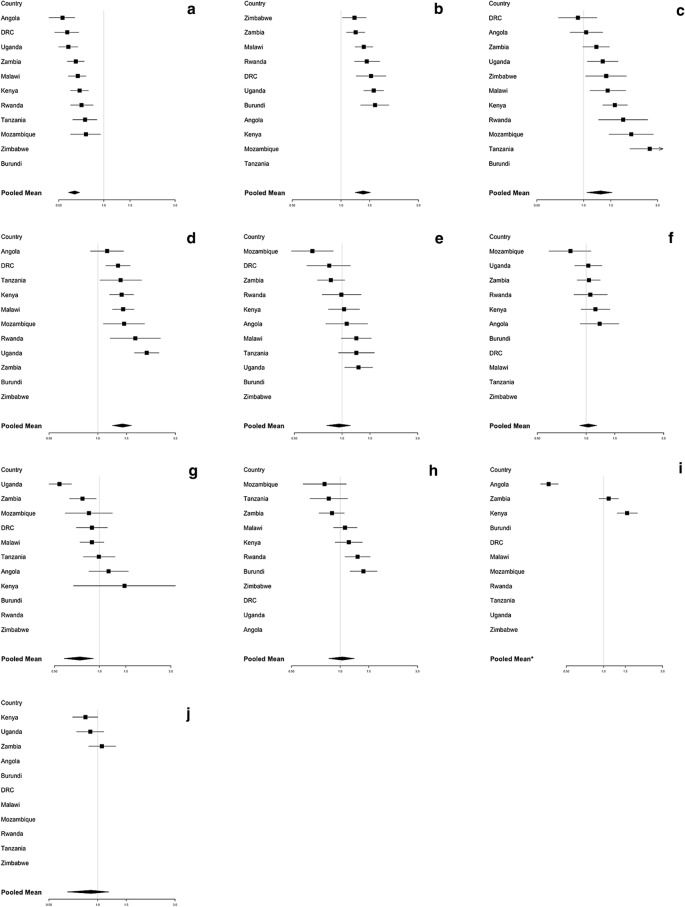
Association between use of ITNs and statistically significant covariate factors. **a** Households with a large number of members (more than 4 people); **b** Households with more than one child under 5; **c** Households located in urban areas; **d** Households with high education level; **e** Houses built with a finished floor material; **f** Houses built with a finished wall material; **g** Houses built with a finished roof material; **h** Wealthy households; **i** Households located in high malaria parasite rate areas (≥ 0.09); **j** Households located in high average land travel per metre areas (≥ 0.003). ^1^*DRC* Democratic Republic of the Congo, *ITN* Insecticide-treated net. ^2^The solid line represents the null value (no difference between groups). ^3^Odds ratios (OR) are adjusted for variables which are significant in unadjusted analyses. ^4^Error bars show 95% confidence intervals

In comparison, households with more than one child under 5 (OR = 1.37; 95% CI 1.22–1.52), households located in urban areas compared to households in rural areas (OR = 1.29; 95% CI 1.05–1.53), and households in which household member had high education (OR = 1.42; 95% CI 1.23–1.61) were all associated with ITN use in households that own ITNS (Fig. 4). Conversely, households with a large number of family members were negatively associated with ITN use in the household (OR = 0.64; 95% CI 0.58–0.69). Additional file 1: Tables S3–S13 in Additional file 1 include all OR estimations.

### Spatiotemporal changes of ITN ownership and use

**Fig. 5:**
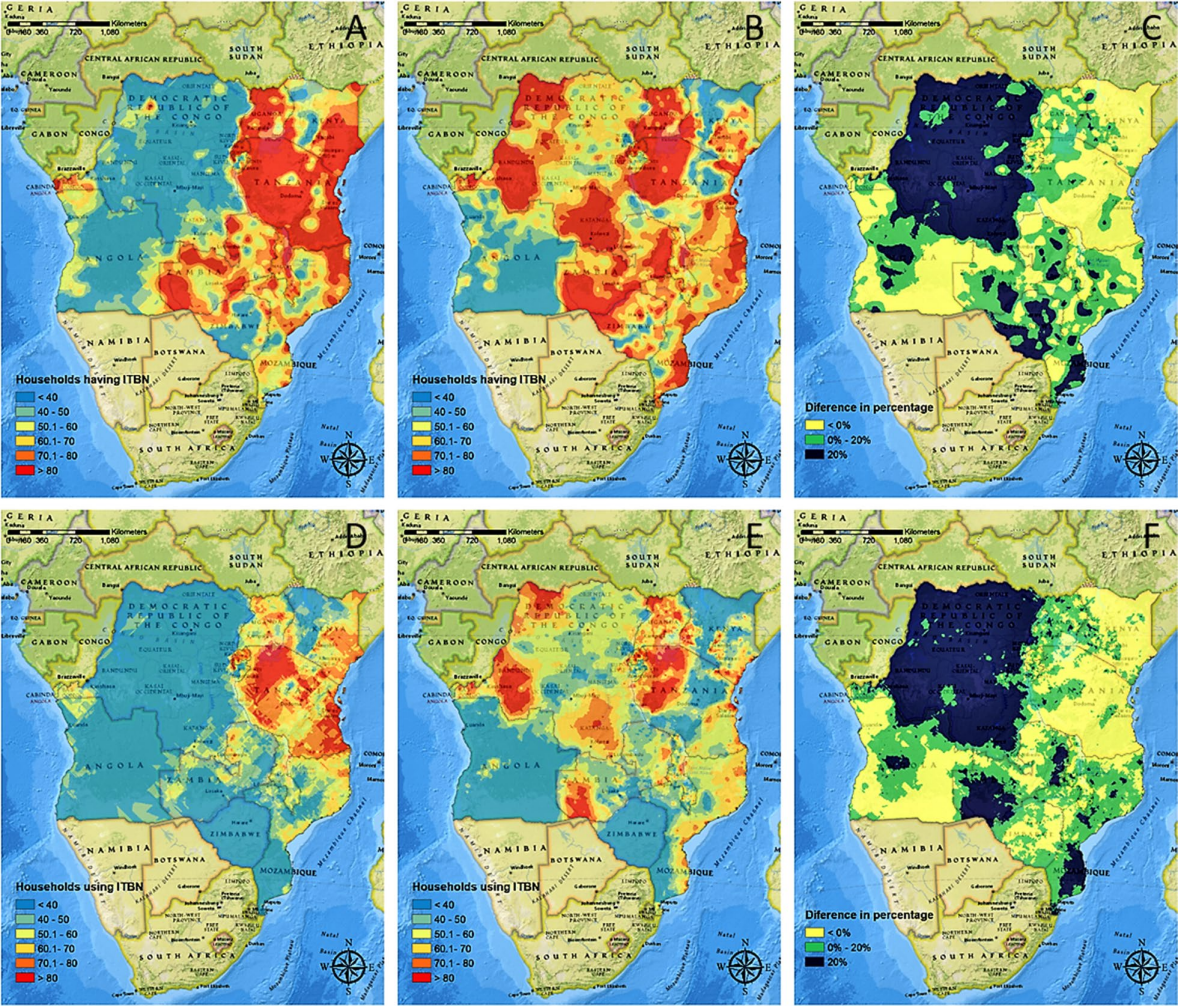
Spatiotemporal dynamics in ownership and use of ITNs between DHS-MIS 2007–2011 and 2013–2017. Prevalence distribution (%) of ITNs *ownership* and *use* of ITNs in DHS-MIS 2007–2011 (**a** and **d**, respectively) and DHS-MIS 2013–2017 (**b** and **e**, respectively). Continuous surface maps of spatial changes between DHS-MIS 2007–2011 and 2013–2017 for ownership (**c**) and use (**f**) of ITNs. Maps were created using ArcGIS^®^ by Esri version 10.5 (http://www.esri.com) [59], and basemaps were obtained from ESRI and National Geographic available at ArcGIS Online basemaps [60]

The spatial distribution of the percentage of households that owned ITNs is illustrated in Fig. 5 for DHS-MIS 2007–2011 (A) and DHS-MIS 2013–2017 (B). For the first DHS wave (DHS-MIS 2007–2011), the spatial analysis indicated that ITN ownership was high in the north-eastern part of the area of study, particularly in Uganda, Rwanda, and Tanzania, where ITN ownership was predominant in more than 60% of the total territory of these countries. In contrast, ITN ownership covered less than 60% of the territory in DRC, Angola, and Zimbabwe (Fig. 5a). For the second DHS wave (DHS-MIS 2013–2017), the territory covered by ITNs ownership increased in all countries except for Angola. The absolute difference in the distribution of the ownership between DHS-MIS 2007–2011 and DHS-MIS 2013–2017 is presented in Fig. 5c, showing the largest changes in the ownership in the north-western part of DRC.

Figure 5d, e illustrate the spatial distribution of the change of ITN use in the two DHS waves. Some countries such as DRC, Kenya, and Tanzania showed substantial spatial heterogeneity of ITN use within each country, with some areas showing higher proportion (> 60%) and some having lower proportion of ITN use (< 40%) for the most recent DHS wave (DHS-MIS 2013–2017). Compared with the previous DHS wave (DHS-MIS 2007–2011), most of the region showed an increment in ITN use except for some areas in the southern and eastern part of the region (Fig. 5f), with negative changes showing in large areas of Angola, Kenya, Tanzania, and Zimbabwe.

### Density map of children under 5 at high-risk of malaria

**Fig. 6:**
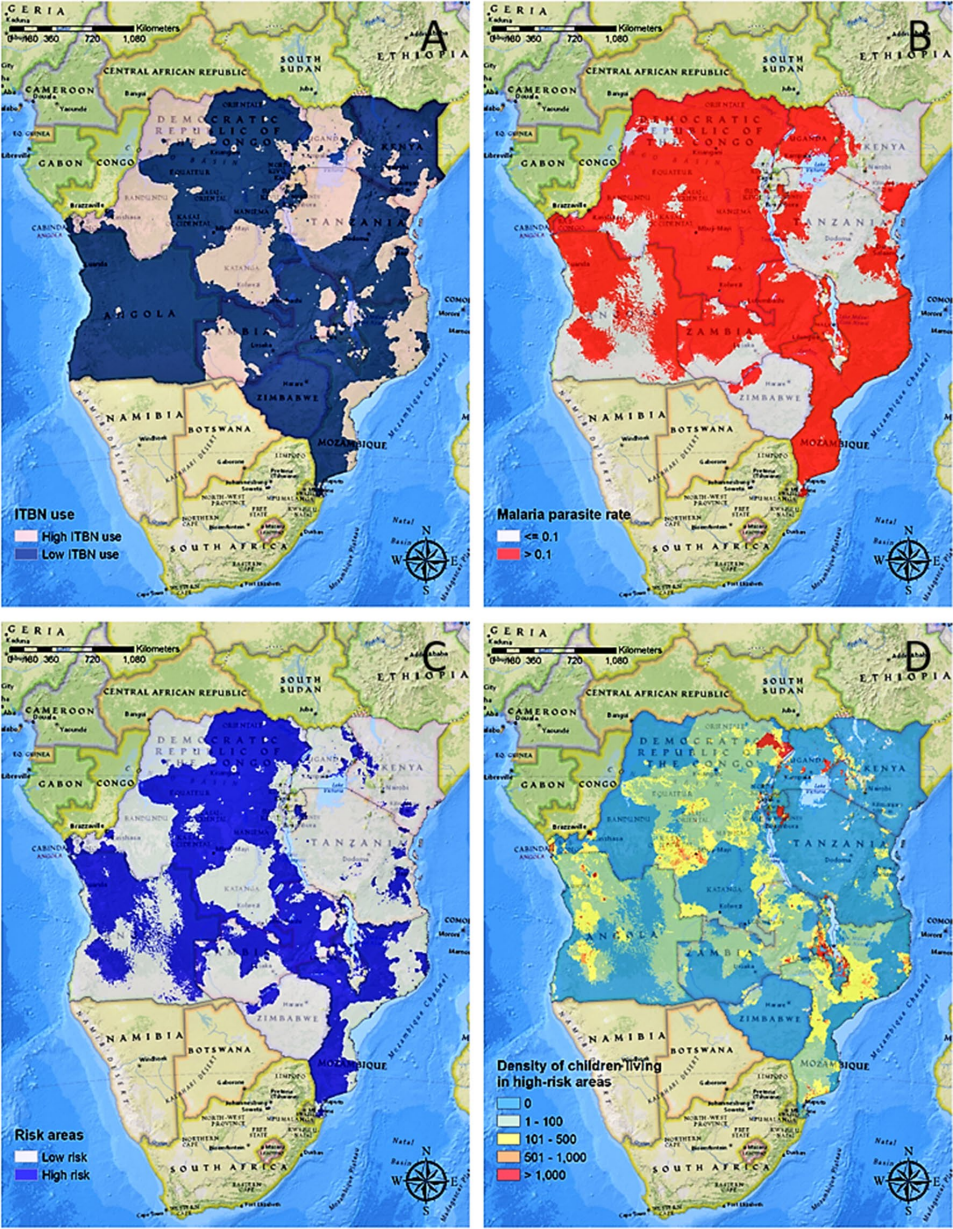
Interpolated surface map of lower (< 60%) and higher levels for use of ITNs (**a**), areas with lower (Parasite Rate, PR < 0.1) and higher endemic malaria (**b**), the combination of both **a** and **b** to identify high-risk areas (low ITNs use and high malaria) (**c**), and the estimated number of children under 5 living at risk of malaria (**d**). Maps were created using ArcGIS^®^ by Esri version 10.5 (http://www.esri.com) [59], and basemaps were obtained from ESRI and National Geographic available at ArcGIS Online basemaps [60]

The map of ITN use estimated from the latest DHS wave (DHS-MIS 2013–2017) (Fig. 6a) and the map of *Pf*PR_2–10_ (Fig. 6b) were combined to estimate the density of children under 5 living in areas with low levels of ITNs use (< 60%) and high *Pf*PR_2–10_ (> 0.1) (Fig. 6d). The analysis indicated that 19,780,678 (23%) of the total population of children under 5 were living in the above-mentioned conditions. In Malawi and Mozambique, more than half of the children under 5 were living in these areas, with 2,600,314 (59%) and 3,928,171 (56%), respectively. In DRC and Angola, a large proportion of children under 5 were also living in low ITN use and high *Pf*PR_2–10_ areas, with 7,843,155 (39%) and 2,359,933 (39%), respectively. In these four countries with low ITN use, the total number of individuals living in the household, education of the head of the household, type of residential place (urban or rural), main floor material, and the number of children under age 5 in the households were significantly associated with ITN use in the household (Additional file 1: Tables S3, S5, S7 and S8).

## Discussion

Analysing data collected from more than 100,000 households in 11 countries in CEA, despite substantial progress in the distribution of ITNs in CEA, with about 70% of the households having an ITN, several socioeconomic factors have compromised the effectiveness of this control intervention against malaria. As a result, only about 48% of the households protect their children under 5 with ITNs. The results suggest that factors such as the number of members in the household, total number of children in the household, education and place of residence can be key factors linked to the use of ITN for protecting children against malaria in CEA. The likelihood of ITN use was higher in smaller households compared to larger households in most countries excluding Burundi and Zimbabwe. This finding concurs with those of recent reviews and analyses of ITN use in African countries including Zambia, and Zimbabwe [7–10], in which the odds of ITN use decreased with larger households, with five or six members compared to those with less than five household members. In terms of the number of children, the results showed that the use of ITNs in households with more than one child under 5 was significantly higher compared to those households with only one child. The reasons for this association have not been well documented and require further investigation. However, a report from Burkina Faso suggests that ITN use is high for households in which a child experienced any illness in the previous 2 weeks. This previous experience of the children’s illness can be a motivation of the use of ITNs [9]. Hence, it is possible that households with more than one child could have higher likelihood of ITN use, based on their experience of previous illness suffered by older children.

Education was another socioeconomic variable linked to ITN use in most of the countries included in this study. The findings suggest that households in which the head of the household had a high education had higher odds of using ITNs. Previous studies have found that misconception about ITN’s usage were linked to lack of education such as lack of knowledge about transmission of malaria and its symptoms, discomfort due to heat within an ITN [42], and the benefits and proper use of ITNs [10–13, 15–18]. The expansion of educational campaigns about malaria transmission and its clinical symptoms, as well as the benefits of ITN use, might play an important role not only in motivating people to use ITN [7, 16], but also in preventing misconceptions and myths and reducing cultural taboos  [17]. Moreover, urban residents were more prone to use ITNs in most countries except in Burundi and DRC. These results are consistent with previous studies in African countries including Guinea, and Ethiopia, in which the households with children under 5 in rural areas were less likely to sleep under an ITN, compared to their urban counterparts  [12, 13]. These studies suggested that the reason for low use of ITN was a difference in attitude or lack of knowledge rather than a problem of access [12], which concurs with the high proportion of ITN ownership but low ITN use in rural areas.

Results from the spatiotemporal analyses found that although total rates of ownership and use of ITNs across CEA have increased up to 70% and 48% respectively, a large proportion of children under 5 (19,780,678; 23% of total number of children) still live in high-risk areas with high burden of malaria but low use of ITNs. From these analyses, countries were identified that have made positive progress but need more attention to develop a stronger policy against malaria. Among the countries included in this study, DRC showed the most impressive results with the highest increase in both ownership and use of ITNs between the surveyed years, 2007 and 2013–2014. In 2011, DRC was selected as a President’s Malaria Initiative focus country, and over 40 million ITNs were distributed in the country [51]. Along with this aggressive campaign of ITN distribution, the results suggest that promoting the use of ITN through education has been a key against malaria. Besides ITN distribution, DRC has focused on education to reduce and prevent malaria through the interpersonal and mass communication strategies such as the national health communication programme, the national school health programme, and community-based organizations [51]. Educational activities that promote social and behavioural changes have been implemented in DRC as a national strategy to promote use of malaria preventive measure and treatment services and to ensure correct and timely use of ITNs in targeted health zones since 2011 [51]. The results observed in DRC highlight the importance of combined programmes of ITN distribution along with informative campaigns that instruct about the modes of malaria transmission, prevention, and promotion of the importance and correct use of ITNs.

In contrast, Tanzania showed negative changes in the use of ITNs in large areas with spatial heterogeneity between the survey years, 2010 and 2015–2016 (67% to 57%, respectively). A previous study in Kigoma region identified a potential reason for the decrease. This region located along Lake Tanganyika showed the second-highest malaria prevalence among children in this country, but most of the respondents in the region (87.2%) reported having ever used ITN for fishing [52]. Although fishing with fine-gauge nets like ITN is illegal, responders declared that they used ITNs because their income was not sufficient to afford proper fishing nets [52]. Likewise, Angola showed slight decreases for both ownership and use of ITNs between the survey years, and both ratios were considerably low (42% in ITNs ownership and 29% ITNs use). The ratio of ITN ownership was the lowest of the countries included in this study. Moreover, around 39% of children under age 5 (2,359,933 children of total) were living in areas with high-risk of malaria, which is the highest rate among the countries included in this study. Angola was selected as one of the first countries in 2005 when the President’s Malaria Initiative was launched, and more than 14 million ITNs have been distributed through various partners since 2006 [53]. Despite the government’s effort, considerable disparities still existed among provinces between urban and rural regarding inhabitants’ access to care and unbalanced distribution of human resources [53, 54]. Moreover, improved housing using finished materials is considered as one of measures for the progress of urbanization [14, 54, 55], and Angola showed a low increase of urbanization while most of CEA countries displayed a greater urban increase [54].

Despite the strengths of this study, several study limitations are worth noting. First, some of the variables included in the study could have been affected by inherent biases in the data, such as overestimation of ITN use. Standard face-to-face household surveys may cause information bias when the respondents feel socially obligated to respond positively to questions about recent ITN use for themselves and their children [6, 56]. For instance, respondents could provide socially desirable answers when some households had potentially misused ITNs, such as for fishing [52]. Likewise, the study did not include environmental factors that could be associated with ITN use, such as seasonality. DHS is conducted every 5 years in different seasons, such as rainy and dry season, and rainfall patterns within and outside of countries are heterogeneous, and environmental factors could have been only partially captured and assessed from the data in the national surveys [6, 7, 16]. Additionally, because the main goal was to identify general geographical patterns of ITN ownership and use in a large geographical area in CEA, ordinary kriging was implemented and did not conduct uncertainty analysis for the spatial model generated in this study. More detailed and accurate maps can be generated using different spatial interpolation methods, such as empirical Bayesian kriging, to consider for uncertainty in the spatial predictions [57]. Lastly, in this study, ITN was considered as the main control intervention against malaria. Other effective control interventions, such as indoor residual spraying, can be used in some of the areas identified as high priority areas [58], potentially resulting in low ITN use in these areas.

## Conclusions

This study suggests that socioeconomic factors, such as the size of household, number of children in the household, urbanization, and education, can be key elements for the success of malaria interventions such as ITN distribution and use for children under 5 in CEA. In addition, increasing the proportion of effective ITN use by targeting these factors can be a core strategy to reduce malaria transmission and to achieve global targets for 2030 of Global Technical Strategy for Malaria.

The study has important implications for global targets for 2030, which have sought to eliminate malaria by achieving milestones for measuring progress by 2025. A modest increase in the ITN use across CEA. However, there is still a considerable gap between ITN ownership and ITN use in CEA. Using spatial epidemiological approaches to investigate geographical distribution of effective use of ITNs, more than 20% of the total population of children under 5 were identified living in areas of high-risk of malaria and low ITN use. These findings highlight the urgent need for not only governments’ actions with intervention policies, but also international attention to promote the use of ITNs for children under 5. Furthermore, this is the first study to provide a comparison between risk factors and use of ITNs across CEA, and the findings can be generalizable across the continent. Therefore, special attention should be given to the development and implementation of tailored prevention programmes, especially in areas identified as high-risk of malaria for children under 5. In the malaria response of each country, statistical modelling and high-resolution maps of at-risk malaria indices provide valuable information in support of a complementary component of the decision-making process to achieve a national and international goal for malaria elimination across CEA. The model results presented in this study provide information aimed at increasing the effective ITN use as a priority intervention in those areas with the greatest need of households living with children under 5, beyond creasing ITN distribution based on the coverage ratio at national or province level.

## Supplementary information

**Additional file 1: Table S1**. Descriptive statistics for households with at least one child age under 5 by country from DHS survey. **Table S2**. Descriptive statistics of socio-behavioral variables included in the analysis. **Table S3**. Results of unadjusted and adjusted models of self-reported ownership and use of ITNs in Angola. **Table S4**. Results of unadjusted and adjusted models of self-reported ownership and use of ITNs in Burundi. **Table S5**. Results of unadjusted and adjusted models of self-reported ownership and use of ITNs in DRC. **Table S6**. Results of unadjusted and adjusted models of self-reported ownership and use of ITNs in Kenya. **Table S7**. Results of unadjusted and adjusted models of self-reported ownership and use of ITNs in Malawi. **Table S8**. Results of unadjusted and adjusted models of self-reported ownership and use of ITNs in Mozambique . **Table S9**. Results of unadjusted and adjusted models of self-reported ownership and use of ITNs in Rwanda. **Table S10**. Results of unadjusted and adjusted models of self-reported ownership and use of ITNs in Tanzania. **Table S11**. Results of unadjusted and adjusted models of self-reported ownership and use of ITNs in Uganda. **Table S12**. Results of unadjusted and adjusted models of self-reported ownership and use of ITNs in Zambia. **Table S13**. Results of unadjusted and adjusted models of self-reported ownership and use of ITNs in Zimbabwe.

## Data Availability

The data that support the findings of this study are available from the Demographic and Health Surveys (http://www.measuredhs.com) but restrictions apply to the availability of these data, which were used under license for the current study, and so are not publicly available. Data are however available from the authors upon reasonable request and with permission of Demographic and Health Surveys.

## Abbreviations

CEA: Central and East Africa
SSA: Sub-Saharan Africa
WHO: World Health Organization
ITN: Insecticide-treated net
DHS: Demographic and health surveys
MIS: Malaria indicator surveys
DRC: Democratic Republic of Congo
PSUs: Primary sample units
*Pf*PR: *Plasmodium falciparum* parasite rate

## References

[1] WHO. World Malaria Report 2018. Geneva, World Health Organization, 2018.

[2] WHO. A framework for malaria elimination. Geneva, World Health Organization, 2017.

[3] WHO. Global technical strategy for malaria 2016–2030. Geneva, World Health Organization, 2015.

[4] Bhatt S, Weiss DJ, Cameron E, Bisanzio D, Mappin B, Dalrymple U (2015). The effect of malaria control on *Plasmodium falciparum* in Africa between 2000 and 2015. Nature.

[5] WHO. Roll Back Malaria Partnership Secretariat. Action and investment to defeat malaria 2016–2030. For a malaria-free world. Geneva, World Health Organization, 2015.

[6] Eisele TP, Keating J, Littrell M, Larsen D, Macintyre K (2009). Assessment of insecticide-treated bednet use among children and pregnant women across 15 countries using standardized national surveys. Am J Trop Med Hyg.

[7] Storey JD, Babalola SO, Ricotta EE, Fox KA, Toso M, Lewicky N (2018). Associations between ideational variables and bed net use in Madagascar, Mali, and Nigeria. BMC Public Health.

[8] Admasie A, Zemba A, Paulos W (2018). Insecticide-treated nets utilization and associated factors among under-5 years old children in Mirab-Abaya District, Gamo-Gofa Zone. Ethiopia. Front Public Health.

[9] Diabaté S, Druetz T, Bonnet E, Kouanda S, Ridde V, Haddad S (2014). Insecticide-treated nets ownership and utilization among under-five children following the 2010 mass distribution in Burkina Faso. Malar J.

[10] Kanyangarara M, Hamapumbu H, Mamini E, Lupiya J, Stevenson JC (2018). Malaria knowledge and bed net use in three transmission settings in southern Africa. Malar J.

[11] García-Basteiro AL, Schwabe C, Aragon C, Baltazar G, Rehman AM, Matias A (2011). Determinants of bed net use in children under five and household bed net ownership on Bioko Island Equatorial Guinea. Malar J.

[12] Baume CA, Franca-Koh AC (2011). Predictors of mosquito net use in Ghana. Malar J.

[13] Graves PM, Ngondi JM, Hwang J, Getachew A, Gebre T, Mosher AW (2011). Factors associated with mosquito net use by individuals in households owning nets in Ethiopia. Malar J.

[14] Tusting LS, Bottomley C, Gibson H, Kleinschmidt I, Tatem AJ, Lindsay SW (2017). Housing improvements and malaria risk in sub-Saharan Africa: a multi-country analysis of survey data. PLoS Med.

[15] Noor AM, Omumbo JA, Amin AA, Zurovac D, Snow RW (2006). Wealth, mother’s education and physical access as determinants of retail sector net use in rural Kenya. Malar J.

[16] Atieli HE, Zhou G, Afrane Y, Lee M-C, Mwanzo I, Githeko AK (2011). Insecticide-treated net (ITN) ownership, usage, and malaria transmission in the highlands of western Kenya. Parasit Vectors.

[17] Ndjinga JK, Minakawa N (2010). The importance of education to increase the use of bed nets in villages outside of Kinshasa, Democratic Republic of the Congo. Malar J.

[18] Ntonifor NH, Veyufambom S (2016). Assessing the effective use of mosquito nets in the prevention of malaria in some parts of Mezam division Northwest Region Cameroon. Malar J.

[19] Koenker H, Kilian A (2014). Recalculating the net use gap: a multi-country comparison of ITN use versus ITN access. PLoS ONE.

[20] Flaxman AD, Fullman N, Otten MW, Menon M, Cibulskis RE, Ng M (2010). Rapid scaling up of insecticide-treated bed net coverage in Africa and its relationship with development assistance for health: a systematic synthesis of supply, distribution, and household survey data. PLoS Med.

[21] Giardina FP, Kasasa SP, Sié AP, Utzinger JP, Tanner MP, Vounatsou PD (2014). Effects of vector-control interventions on changes in risk of malaria parasitaemia in sub-Saharan Africa: a spatial and temporal analysis. Lancet Glob Health.

[22] Instituto Nacional de Estatística INEA, Minstério da Saúde MA, ICF. Angola Inquérito de Indicadores Múltiplos e de Saúde (IIMS) 2015–2016. Luanda, INE, MINSA, and ICF, 2017.

[23] Ministère à la Présidence chargé de la Bonne Gouvernance et du Plan M, Ministère de la Santé Publique et de la Lutte contre le Sida M, Institut de Statistiques et d’Études Économiques du Burundi I, ICF. Burundi Troisième Enquête Démographique et de Santé 2016–2017. Bujumbura, MPBGP, MSPLS, ISTEEBU, and ICF, 2017.

[24] Ministère du Plan et Suivi de la Mise en œuvre de la Révolution de la Modernité–MPSMRM/Congo, Ministère de la Santé Publique–MSP/Congo, ICF. République Démocratique du Congo Enquête Démographique et de Santé (EDS-RDC) 2013–2014. Rockville, MPSMRM, MSP, and ICF, 2014.

[25] Kenya National Bureau of S, Ministry of HK, National ACCK, Kenya Medical Research I, National Council for Population and DK. Kenya Demographic and Health Survey 2014. Rockville, 2015.

[26] National Statistical OM, ICF. Malawi Demographic and Health Survey 2015–2016. Zomba, National Statistical Office, ICF, 2017.

[27] Ministério da Saúde M, Nacional Instituto, de Estatística INE (2015). ICF. Inquérito de Indicadores de Imunização, Malária e HIV, SIDA em Moçambique.

[28] National Institute of Statistics of R, Ministry of Finance and Economic PR, Ministry of HR, ICF. Rwanda Demographic and Health Survey 2014–15. Kigali, National Institute of Statistics of Rwanda, Ministry of Finance and Economic Planning/Rwanda, Ministry of Health/Rwanda, ICF, 2016.

[29] Ministry of Health CDGEaCMTM, Ministry of Health–Mo HZ, National Bureau of Statistics NBST, Office of Chief Government Statistician OZ, ICF. Tanzania Demographic and Health Survey and Malaria Indicator Survey 2015–2016. Dar es Salaam, MoHCDGEC, MoH, NBS, OCGS, ICF, 2016.

[30] Uganda Bureau of Statistics U, ICF. Uganda Demographic and Health Survey (2016). Kampala.

[31] Central Statistical OZ, Ministry of HZ, University of Zambia Teaching Hospital Virology L, University of Zambia Department of Population S, Tropical Diseases Research CZ, ICF. Zambia Demographic and Health Survey 2013–2014. Rockville, Central Statistical Office/Zambia, Ministry of Health/Zambia, ICF, 2015.

[32] Zimbabwe National Statistics A, ICF. Zimbabwe Demographic and Health Survey 2015: Final Report. Rockville, Zimbabwe National Statistics Agency, ICF, 2016.

[33] Croft TN, Marshall AMJ, Allen CK (2018). Guide to DHS Statistics.

[34] Hay SI, Guerra CA, Gething PW, Patil AP, Tatem AJ, Noor AM (2009). A world malaria map: *Plasmodium falciparum* endemicity in 2007. PLoS Med.

[35] Weiss DJ, Nelson A, Gibson HS, Temperley W, Peedell S, Lieber A (2018). A global map of travel time to cities to assess inequalities in accessibility in 2015. Nature.

[36] Malaria Atlas Project (MAP). https://map.ox.ac.uk/.

[37] Kelly C, Hulme C, Farragher T, Clarke G (2016). Are differences in travel time or distance to healthcare for adults in global north countries associated with an impact on health outcomes? A systematic review. BMJ Open.

[38] ESRI: ArcGIS 10.x. Redlands, ESRI, 2004.

[39] Demographic Health Survey (DHS) Official Website [https://dhsprogram.com/].

[40] Riley RD, Higgins JPT, Deeks JJ (2011). Interpretation of random effects meta-analyses. BMJ.

[41] Team RDC: R: A Language and Environment for Statistical Computing. 2008.

[42] Inc. SI: SAS software. 2018.

[43] Carrat F, Valleron A-J (1992). Epidemiologic mapping using the “kriging” method: application to an influenza-like epidemic in France. Am J Epidemiol.

[44] Berke O (2004). Exploratory disease mapping: kriging the spatial risk function from regional count data. Int J Health Geogr.

[45] Goovaerts P (2005). Geostatistical analysis of disease data: estimation of cancer mortality risk from empirical frequencies using Poisson kriging. Int J Health Geogr.

[46] Naish S, Dale P, Mackenzie JS, McBride J, Mengersen K, Tong S (2014). Spatial and temporal patterns of locally-acquired dengue transmission in northern Queensland, Australia, 1993–2012. PLoS ONE.

[47] Oliver MA, Webster R (1990). Kriging: a method of interpolation for geographical information systems. Int J Geogr Inf Sci.

[48] Linard C, Gilbert M, Snow RW, Noor AM, Tatem AJ (2012). Population distribution, settlement patterns and accessibility across Africa in 2010. PLoS ONE.

[49] WorldPop Project [https://www.worldpop.org].

[50] Africa ECF. The Demographic Profile of African Countries. 2016.

[51] PMI. Malaria Operational Plan FY 2018: Democratic Republic of the Congo. 2018.

[52] McLean KA, Byanaku A, Kubikonse A, Tshowe V, Katensi S, Lehman AG (2014). Fishing with bed nets on Lake Tanganyika: a randomized survey. Malar J.

[53] PMI. Malaria Operational Plan FY 2016: Angola. 2016.

[54] Tatem AJ, Gething PW, Smith DL, Hay SI (2013). Urbanization and the global malaria recession. Malar J.

[55] Tusting LS, Bisanzio D, Alabaster G, Cameron E, Cibulskis R, Davies M (2019). Mapping changes in housing in sub-Saharan Africa from 2000 to 2015. Nature.

[56] Skarbinski J, Winston CA, Massaga JJ, Kachur SP, Rowe AK (2008). Assessing the validity of health facility-based data on insecticide-treated bednet possession and use: comparison of data collected via health facility and household surveys–Lindi region and Rufiji district, Tanzania, 2005. Trop Med Int Health.

[57] Krivoruchko K. Empirical bayesian kriging. ESRI: Redlands, CA. https://www.esri.com/news/arcuser/1012/empirical-byesian-kriging.html [Verified October 2018] 2012.

[58] Pluess B, Tanser FC, Lengeler C, Sharp BL (2010). Indoor residual spraying for preventing malaria. Cochrane Database Syst Rev.

[59] ESRI. ArcGIS 10.x. Redlands, CA, ESRI. 2004.

[60] ESRI. “Topographic” [basemap]. “World Topographic Map”. 2012. http://www.arcgis.com/home/item.html?id=30e5fe3149c34df1ba922e6f5bbf808f.

