# Supplementary material for: Spatiotemporal analysis of insecticide-treated net use for children under 5 in relation to socioeconomic gradients in Central and East Africa
Source: Malar J. 2020 Apr 22;19:163. doi: 10.1186/s12936-020-03236-2 (PMC7178571; doi:10.1186/s12936-020-03236-2)
Supplement: Supplementary file 1 — Additional file 1: Table S1. Descriptive statistics for households with at least one child age under 5 by country from DHS survey. Table S2. Descriptive statistics of socio-behavioral variables included in the analysis. Table S3. Results of unadjusted and adjusted models of self-reported ownership and use of ITNs in Angola. Table S4. Results of unadjusted and adjusted models of self-reported ownership and use of ITNs in Burundi. Table S5. Results of unadjusted and adjusted models of self-reported ownership and use of ITNs in DRC. Table S6. Results of unadjusted and adjusted models of self-reported ownership and use of ITNs in Kenya. Table S7. Results of unadjusted and adjusted models of self-reported ownership and use of ITNs in Malawi. Table S8. Results of unadjusted and adjusted models of self-reported ownership and use of ITNs in Mozambique . Table S9. Results of unadjusted and adjusted models of self-reported ownership and use of ITNs in Rwanda. Table S10. Results of unadjusted and adjusted models of self-reported ownership and use of ITNs in Tanzania. Table S11. Results of unadjusted and adjusted models of self-reported ownership and use of ITNs in Uganda. Table S12. Results of unadjusted and adjusted models of self-reported ownership and use of ITNs in Zambia. Table S13. Results of unadjusted and adjusted models of self-reported ownership and use of ITNs in Zimbabwe. [file 12936_2020_3236_MOESM1_ESM.pdf]

Supplementary Materials for:

Spatiotemporal analysis of insecticide-treated net use for children under 5 in relation to socioeconomic gradients in Central and East Africa

Hana Kim, F. DeWolfe Miller, Frank Tanser,

Polycarp Mogeni, Diego F. Cuadros*

* Reprints or correspondence: Diego F. Cuadros, PhD, E-mail: diego.cuadros@uc.edu. Department of Geography and Geographic Information Science, University of Cincinnati, Cincinnati, OH, 45221. Telephone: (513) 556-3423. Fax: (513) 556-3370.

Supplementary methods

Definition of socio-economic and behavioral variables

To assess the association between socio-economic and behavioral factors and the prevalence of ownership and use of ITNs, we selected a variety of demographic, socio-economic, and behavioral attributes from the DHS dataset. Eight variables measured for households were included: total number of household members, total number of children aged under 5 in the household, type of place of residence (urban and rural), highest level of education the household member attended, housing qualities (main floor, wall, and roof materials of the house), and wealth index. Total house member of household members counts not only the number of usual residents but also the number of visitors who slept in the house the previous night that the night before the survey schedule [1]. The value of total number of household members of eleven countries were combined to calculate median. Based on the median (5 people), total number of household members were sorted into two categories containing an equal number of values: *small size of household (less than 5 people)* and *large size of household (greater than or equal to 5 people)*. Number of children in the households contains children resident in the household and aged 5 and under, and visiting children are not counted [1]. In this analysis, the variable was sorted into two levels: *only child* and *more than two children.* We did not reclassify the values of the type of place of residence of DHS questionnaire, which contains two values: *urban* and *rural* [1]. Level of education is from the question that the highest level of education the household member attended. DHS standardized variable into *No education, Primary, Secondary, and Higher* [1]. We reclassified into binary: *Low* and *High*. *Low* category contains households with no education or primary education while *high* category contains household with secondary or higher education.

Housing variables used in the analysis were the main materials used for the construction of floors, walls, and roofs among the surveyed households [2]. Each housing material is divided into categories of natural, rudimentary, and finished. Natural or rudimentary materials commonly include palm, wood planks, and bamboo, while finished materials typically include cement, (polished) wood, and ceramic tiles [1]. In order to identify impact of the finished materials, we used two categories: *unfinished and finished*. *Unfinished* category contains households whose main housing material is either natural or rudimentary. *Finished* category contains households whose main housing material is finished. Wealth index is an ordinal variable that describes standard of living as determined by material possessions. The DHS collected and calculated responses to questions associated with wealth status, such as an ownership of a television and car, housing construction materials, type of drinking water source, and access to sanitation facilities [1, 3]. This index was classified into two categories: *poor and wealthy*. *Poor* category contains poorest and poorer household, and *wealthy* category contains middle, richer, and richest household.

Definition of malaria endemicity and transmission

We used a *Plasmodium falciparum* parasite rate (*Pf*PR) among children aged 2-10 years as a malaria covariate to identify exposure to malaria infection in 2-10 years old. *Plasmodium falciparum* parasite is a vector that transmits malaria and is used as an endemicity and transmission intensity indicator [4, 5]. Malaria Atlas Project (MAP) has been generating P. *falciparum* endemicity based on evidence-based estimates of populations at malaria risk [6, 7]. As of July 2018, the MAP contains nearly 49,290 clusters for *P.falciparum* across 87 malaria-endemic countries. The $Pf\mathrm{PR}_{2\text{-}10}$ from MAP is age standardized to 2-10 years old and is generated on a continuous surface as a form of raster data after implementing geostatistical algorithms for filling spatial gaps.

As a transmission variable, an average land travel speed, is obtained from a global friction surface of the MAP to represent spatial accessibility of each Primary Sample Unit (PSU). The optimal speed of travel computed, and friction surface represents allocated fastest speed of travel based on the types of travel mode occurring within the pixel. Friction map used mainly the most updated data until 2015 for road, railroad and water travel distances from the Open Street Maps (OMS) and Google roads projects. The datasets include roads, railways, rivers, lakes, oceans, topographic conditions (e.g., slope and elevation), landcover types, and national borders [8]. For the portions of land that did not covered by any of above-mentioned dataset, a baseline speed of movement on foot was derived using the MODIS MCD12Q1 land cover product [9]. Land cover speeds were adjusted to take into account the effect of topographical properties (elevation, slope, and Tobler's walking speed); then, urban land cover dataset from the Global Human Settlement project was overlapped to represent urban areas [8]. Values for these two variables were extracted at each PSU location by overlaying PSU location using ArcGIS and were used to calculate medians. Based on each median value (0.09 for *Pf*PR and 0.003 for average land travel speed), each value was classified into binary value (high and low) and assigned to each household.

Definition of environmental variables

To map the density of children under age 5 at high risk of malaria, we used estimates of the 2015 population density throughout the countries from the WorldPop database. Contemporary population count data with detailed satellite-derived settlement extents was resampled to 100m X 100m resolution is used to generate population distribution. Urban areas replaced with the expanded surrounding classes because of the overestimated settlement extent size. Then, the population density was generated by combining population of major settlement and redistributed rural population using land cover-based weights from the population distribution [10, 11]. Estimates of population that we used in the analysis were as a raster image with a resolution of 100m x 100m pixel. For consistency with the resolution of the maps generated previously, we reduced the resolution of the raster file to 5km x 5km pixel. Population density map for children

under 5 in the study area was generated using estimates from the Demographic Profile of African Countries report [12].

Supplementary tables

Supplementary Table 1. Descriptive statistics for households with at least one child age under 5 by country from DHS survey.

	AO	BU	DRC	KE	MW	MZ	RW	TZ	UG	ZM	ZW
The number of households											
of the country	16,109	15,977	18,171	36,430	26,361	7,169	12,699	12,563	19,588	15,920	10,534
with at least one child under 5 (%)	9,845 (61)	9,394 (59)	11,624 (64)	16,795 (46)	15,180 (58)	4,037 (56)	6,586 (52)	7,321 (58)	11,345 (58)	9,911 (62)	5,462 (52)
% of household with											
one child	40.57	47.30	38.38	58.02	65.18	54.92	63.86	52.97	48.77	49.60	65.65
low education level	59.45	87.33	42.97	69.43	71.08	76.76	87.97	82.54	69.60	55.41	36.31
unfinished floor material	59.67	81.01	86.52	63.97	75.18	61.61	77.16	59.06	70.39	61.50	27.46
unfinished wall material	65.27	42.62	75.37	58.83	22.92	65.92	70.35	23.06	64.79	31.64	16.26
unfinished roof material	14.09	16.53	70.84	96.36	55.72	52.02	0.29	27.31	35.42	49.40	28.49
low income	50.62	41.39	49.10	51.19	42.08	34.95	44.52	38.49	46.29	44.20	38.14
% of malaria	14.00	27.00	23.00	5.00	24.00	40.00	2.00	7.00	30.00	21.00	14.00

Supplementary Table 2. Descriptive statistics of socio-behavioral variables included in the analysis

	AO<br>Count (%)	BU<br>Count (%)	DRC<br>Count (%)	KE<br>Count (%)	MW<br>Count (%)	MZ<br>Count (%)
Total population	9,845	9,394	11,624	16,795	15,180	4,037
Total number of household members						
Small (<5)	4,938 (50.16)	4,605 (49.02)	4,906 (42.21)	9,319 (55.49)	8,826 (58.14)	2,111 (52.29)
Large (≥5)	4,907 (49.84)	4,789 (50.98)	6,718 (57.79)	7,476 (44.51)	6,354 (41.86)	1,926 (47.71)
Total number of children in the household						
1	3,994 (40.57)	4,443 (47.30)	4,461 (38.38)	9,745(58.02)	9,895(65.18)	2,217 (54.92)
2 or more	5,851 (59.43)	4,951 (52.70)	7,163 (61.62)	7,050(41.98)	5,285(34.82)	1,820 (45.08)
Type of place of residence						
Rural	4,292 (43.60)	7,808(83.12)	8,169(70.28)	11,205 (66.72)	12,626 (83.18)	2,444 (60.54)
Urban	5,553 (56.40)	1,586 (16.88)	3,455 (29.72)	5,590 (33.28)	2,554 (16.82)	1,593 (39.46)
Highest educational level						
Low	5,853 (59.45)	8,204 (87.33)	4,995 (42.97)	11,660 (69.43)	10,790 (71.08)	3,099 (76.76)
High	3,784 (38.44)	1,184 (12.6)	6,610 (56.87)	5,088 (30.29)	4,282 (28.21)	908 (22.49)
NA						30 (0.74)
Main floor material						
Unfinished	5,853 (59.45)	8,204 (87.33)	4,995 (42.97)	11,660 (69.43)	10,790 (71.08)	2,487 (61.61)
Finished	3,784 (38.44)	1,184 (12.60)	6,610 (56.87)	5,088 (30.29)	4,282 (28.21)	1,543 (38.22)
NA	- (0)	- (0)	3 (0.03)	4 (0.02)	- (0)	8 (0.20)
Main wall material						
Unfinished	6,426 (65.27)	4,004 (42.62)	8,761 (75.37)	9,880 (58.83)	3,479 (22.92)	2,661 (65.92)
Finished	3,409 (34.63)	5,329 (56.73)	2,683 (23.08)	6,535 (38.91)	11,700 (77.08)	1,367 (33.86)
NA	8 (0.08)	180 (1.92)	180 (1.55)	380 (2.26)	- (0)	8 (0.20)
Main roof material						
Unfinished	1,387 (14.09)	1,553 (16.53)	8,234 (70.84)	16,184 (96.36)	8,458 (55.72)	2,100 (52.02)
Finished	8,410 (85.42)	7,826 (83.31)	3,373 (29.02)	324 (1.93)	6,719 (44.26)	1,922 (47.88)
NA	23 (0.23)	15 (0.16)	17 (0.15)	287 (1.71)	2 (0.01)	4 (0.10)
Have a ITN for sleeping						

No	5,677 (57.66)	4,084 (43.47)	3,148 (27.08)	4,760 (28.34)	4,434 (29.21)	989 (24.50)
Yes	4,168 (42.34)	5,310 (56.53)	8,473 (72.89)	12,032 (71.64)	10,746 (70.79)	3,048 (75.50)
NA	- (0)	- (0)	3 (0.03)	3 (0.02)	- (0)	- (0)
Children under 5 slept using ITN last night						
No	6,344 (64.44)	4,300 (45.77)	4,158 (35.77)	6,177 (36.78)	6,193 (40.80)	1,646 (40.77)
Yes	2,860 (29.05)	4,452 (47.39)	6,761 (58.16)	8,845 (52.66)	7,431 (48.95)	2,121 (52.54)
NA	641 (6.51)	642 (6.83)	705 (6.07)	1,773 (10.56)	1,556 (10.25)	270 (6.69)
Wealth index						
Poor	4,984 (50.62)	3,888 (41.39)	5,707 (49.1)	8,597 (51.19)	6,387 (42.08)	1,411 (34.95)
Wealthy	4,861 (49.38)	5,506 (58.61)	5,917 (50.9)	8,198 (48.81)	8,793 (57.92)	2,626 (65.05)
Malaria parasite rate						
Low (<0.09)	9,833 (99.88)	4,036 (42.96)	1,935 (16.65)	6,640 (39.54)	3,183 (20.97)	230 (5.70)
High (≥0.09)	12 (0.12)	4,409 (46.93)	8,497 (73.10)	8,180 (48.70)	11,997 (79.03)	3,807 (94.30)
NA	- (0)	949 (10.10)	1,192 (10.25)	1,975 (11.76)	- (0)	- (0)
Average land travel friction per meter						
Low (<0.003)	- (0)	6,151 (65.48)	5,537 (47.63)	10,533 (62.72)	10,128 (66.72)	1,943 (48.13)
High (≥0.003)	9,731 (98.84)	3,243 (34.52)	6,087 (52.37)	6,262 (37.28)	5,052 (33.28)	2,094 (51.87)
NA	114 (1.16)	- (0)	- (0)	- (0)	- (0)	- (0)

	RW<br>Count (%)	TZ<br>Count (%)	UG<br>Count (%)	ZM<br>Count (%)	ZW<br>Count (%)
Total population	6,586	7,321	11,345	9,911	5,462
Total number of household members					
Small (<5)	4,183 (63.51)	3,322 (45.38)	5,759 (50.76)	3,465 (34.96)	2,316 (42.40)
Large (≥5)	2,403 (36.49)	3,999 (54.62)	5,586 (49.24)	6,446 (65.04)	3,146 (57.60)
Total number of children in the household					
1	4,206 (63.86)	3,878 (52.97)	5,533 (48.77)	4,916 (49.6)	3,586 (65.65)
2 or more	2,380 (36.14)	,3443 (47.03)	5,812 (51.23)	4,995 (50.4)	1,876 (34.35)
Type of place of residence					
Rural	5,203 (79.00)	,5431 (74.18)	9,144 (80.6)	5,911 (59.64)	3,450 (63.16)
Urban	1,383 (21.00)	1,890 (25.82)	2,201 (19.4)	4,000 (40.36)	2,012 (36.84)
Highest educational level					
Low	5,794 (87.97)	6,043 (82.54)	7,896 (69.60)	5,492 (55.41)	1,983 (36.31)
High	789 (11.98)	1,270 (17.35)	3,303 (29.11)	4,372 (44.11)	3,435 (62.89)
NA	3 (0.05)	8 (0.11)	146 (1.29)	47 (0.47)	44 (0.81)
Main floor material					
Unfinished	5,082 (77.16)	4,324 (59.06)	7,986 (70.39)	6,095 (61.50)	1,500 (27.46)
Finished	1,495 (22.7)	2,989 (40.83)	3,346 (29.49)	3,811 (38.45)	3,952 (72.35)
NA	2 (0.03)	- (0)	- (0)	4 (0.04)	- (0)
Main wall material					
Unfinished	4,633 (70.35)	1,688 (23.06)	7,350 (64.79)	3,136 (31.64)	888 (16.26)
Finished	1,948 (29.58)	5,550 (75.81)	3,972 (35.01)	6,724 (67.84)	4,562 (83.52)
NA	5 (0.08)	83 (1.13)	17 (0.15)	50 (0.50)	8 (0.15)
Main roof material					
Unfinished	19 (0.29)	1,999 (27.31)	4,018 (35.42)	4,896 (49.4)	1,556 (28.49)
Finished	6,567 (99.71)	5,316 (72.61)	7317 (64.5)	5,004 (50.49)	3,900 (71.4)
NA	- (0)	6 (0.08)	4 (0.04)	10 (0.10)	2 (0.04)
Have a ITN for sleeping					
No	1,049 (15.93)	1,705 (23.29)	2,060 (18.16)	2,335 (23.56)	1,924 (35.23)
Yes	5,537 (84.07)	5,616 (76.71)	9,285 (81.84)	7,576 (76.44)	3,538 (64.77)

NA	- (0)	- (0)	- (0)	- (0)	- (0)
Children under 5 slept using ITN last night					
No	1,678 (25.48)	2,346 (32.04)	3,285 (28.96)	4,415 (44.55)	4,075 (74.61)
Yes	4,285 (65.06)	4,208 (57.48)	7,039 (62.04)	4,627 (46.69)	700 (12.82)
NA	623 (9.46)	767 (10.48)	1,021 (9.00)	869 (8.77)	687 (12.58)
Wealth index					
Poor	2,932 (44.52)	2,818 (38.49)	5,252 (46.29)	4,381 (44.2)	2,083 (38.14)
Wealthy	3,654 (55.48)	4,503 (61.51)	6,093 (53.71)	5,530 (55.8)	3,379 (61.86)
Malaria parasite rate					
Low (<0.09)	5,159 (78.33)	6,041 (82.52)	1,885 (16.62)	3,068 (30.96)	5,277 (96.61)
High (≥0.09)	82 (1.25)	1161 (15.86)	8,926 (78.68)	6,814 (68.75)	185 (3.39)
NA	1,345 (20.42)	119 (1.63)	534 (4.71)	29 (0.29)	- (0)
Average land travel friction per meter					
Low (<0.003)	4,388 (66.63)	3,797 (51.86)	4,355 (38.39)	3,101 (31.29)	1,080 (19.77)
High (≥0.003)	2,198 (33.37)	3,524 (48.14)	6,990 (61.61)	6,810 (68.71)	4,382 (80.23)
NA	- (0)	- (0)	- (0)	- (0)	- (0)

Supplementary Table 3. Results of unadjusted and adjusted models of self-reported ownership and use of ITNs in **Angola**. [1, 2]

	**Ownership of ITNs**		**Use of ITNs**	
	Unadjusted OR (95% CI)	Adjusted OR (95% CI)	Unadjusted OR (95% CI)	Adjusted OR (95% CI)
Total number of household members				
Small (<5)	Ref.		Ref.	Ref.
Large (≥5)	0.99 (0.88-1.12)		0.54 (0.45-0.66)	**0.53***** (0.43-0.64)
Total number of children in the household				
1	Ref.		Ref.	
2 or more	1.10 (0.98-1.24)		1.07 (0.89-1.30)	
Type of place of residence				
Rural	Ref.		Ref.	Ref.
Urban	1.11 (0.93-1.33)		1.25 (1.01-1.55)	1.04 (0.82-1.33)
Highest educational level				
Low	Ref.	Ref.	Ref.	Ref.
High	1.36(1.18-1.57)	**1.26**** (1.09-1.46)	1.33 (1.09-1.62)	1.14 (0.90-1.44)
Main floor material				
Unfinished	Ref.		Ref.	Ref.
Finished	1.03 (0.88-1.20)		1.26 (1.01-1.58)	**1.07***** (0.79-1.45)
Main wall material				
Unfinished	Ref.		Ref.	Ref.
Finished	0.92 (0.79-1.07)		1.33 (1.06-1.66)	**1.21***** (0.92-1.59)
Main roof material				
Unfinished	Ref.	Ref.	Ref.	Ref.
Finished	1.89 (1.51-2.37)	**1.74***** (1.39-2.18)	1.36 (1.01-1.81)	**1.15***** (0.85-1.56)
Wealth index				
Poor	Ref.		Ref.	
Wealthy	1.17 (0.99-1.11)		1.17 (0.95-1.44)	
Malaria parasite rate				
Low (<0.09)	Ref.		Ref.	Ref.
High (≥0.09)	1.02 (0.94-1.11)		0.54 (0.49-0.60)	**0.36***** (0.31-0.43)
Average land travel friction per meter				
Low (<0.003)	Ref.			
High (≥0.003)	-			

[1] Boldfaced numbers indicate statistical significance by confidence interval

[2] ‘*’ symbol represents p-values (* $p < 0.05$, ** $p < 0.01$, *** $p < 0.001$)

Supplementary Table 4. Results of unadjusted and adjusted models of self-reported ownership and use of ITNs in **Burundi**. [1, 2]

	**Ownership of ITNs**		**Use of ITNs**	
	Unadjusted OR (95% CI)	Adjusted OR (95% CI)	Unadjusted OR (95% CI)	Adjusted OR (95% CI)
Total number of household members				
Small (<5)	Ref.		Ref.	
Large (≥5)	1.04 (0.94-1.14)		1.00 (0.83-1.20)	
Total number of children in the household				
1	Ref.	Ref.	Ref.	Ref.
2 or more	1.25 (1.15-1.37)	**1.29***** (1.18-1.42)	1.61 (1.32-1.97)	**1.62***** (1.32-1.97)
Type of place of residence				
Rural	Ref.	Ref.	Ref.	
Urban	2.66 (2.03-3.48)	0.95 (0.81-1.11)	1.32 (0.95-1.85)	
Highest educational level				
Low	Ref.	Ref.	Ref.	
High	3.03 (2.54-3.61)	**2.23***** (1.81-2.74)	1.31 (0.96-1.80)	
Main floor material				
Unfinished	Ref.		Ref.	
Finished	2.30 (1.94-2.73)	1.11 (0.91-1.35)	1.16 (0.88-1.53)	
Main wall material				
Unfinished	Ref.	Ref.	Ref.	
Finished	1.84 (1.66-2.06)	**1.15*** (1.02-1.31)	1.02 (0.84-1.24)	
Main roof material				
Unfinished	Ref.	Ref.	Ref.	
Finished	2.20 (1.91-2.53)	**1.30***** (1.11-1.53)	1.03 (0.87-1.38)	
Wealth index				
Poor	Ref.	Ref.	Ref.	Ref.
Wealthy	2.37 (2.13-2.64)	**1.67***** (1.47-1.88)	1.38 (1.14-1.68)	**1.39***** (1.15-1.69)
Malaria parasite rate				
Low (<0.09)	Ref.	Ref.	Ref.	
High (≥0.09)	0.62 (0.53-0.71)	**0.83*** (0.71-0.97)	1.09 (0.86-1.38)	
Average land travel friction per meter				
Low (<0.003)	Ref.	Ref.	Ref.	
High (≥0.003)	0.83 (0.72-0.96)	0.95 (0.81-1.11)	0.91 (0.73-1.15)	

[1] Boldfaced numbers indicate statistical significance by confidence interval

[2] '*' symbol represents p-values (* $p < 0.05$, ** $p < 0.01$, *** $p < 0.001$)

Supplementary Table 5. Results of unadjusted and adjusted models of self-reported ownership and use of ITNs in **DRC**. [1, 2]

	**Ownership of ITNs**		**Use of ITNs**	
	Unadjusted OR (95% CI)	Adjusted OR (95% CI)	Unadjusted OR (95% CI)	Adjusted OR (95% CI)
Total number of household members				
Small (<5)	Ref.		Ref.	Ref.
Large (≥5)	1.11 (0.97-1.26)		0.64 (0.54-0.75)	**0.57*** ** (0.47-0.68)
Total number of children in the household				
1	Ref.		Ref.	Ref.
2 or more	1.06 (0.93-1.20)		1.32 (1.10-1.60)	**1.53*** ** (1.24-1.89)
Type of place of residence				
Rural	Ref.		Ref.	
Urban	1.20 (0.96-1.51)		0.84 (0.68-1.03)	
Highest educational level				
Low	Ref.	Ref.	Ref.	Ref.
High	1.82 (1.57-2.10)	**1.68*** ** (1.44-1.94)	1.25 (1.05-1.49)	**1.33** ** (1.12-1.58)
Main floor material				
Unfinished	Ref.		Ref.	Ref.
Finished	1.03 (0.81-1.31)		0.78 (0.60-0.83)	0.83 (0.60-1.13)
Main wall material				
Unfinished	Ref.		Ref.	
Finished	1.13 (0.92-1.38)		0.81 (0.66-1.01)	
Main roof material				
Unfinished	Ref.		Ref.	Ref.
Finished	1.12 (0.93-1.37)		0.82 (0.67-0.99)	0.89 (0.70-1.13)
Wealth index				
Poor	Ref.	Ref.	Ref.	
Wealthy	1.60 (1.34-1.91)	**1.34** (**1.12-1.61)	1.00 (0.83-1.20)	
Malaria parasite rate				
Low (<0.09)	Ref.	Ref.	Ref.	
High (≥0.09)	0.50 **(**0.34-0.73)	**0.51*** (0.35-0.75)	0.99 (0.72-1.35)	
Average land travel friction per meter				
Low (<0.003)	Ref.		Ref.	
High (≥0.003)	0.91 (0.70-1.18)		1.05 (0.84-1.31)	

[1] Boldfaced numbers indicate statistical significance by confidence interval

[2] '*' symbol represents p-values (* p < 0.05, ** p < 0.01, *** p < 0.001)

Supplementary Table 6. Results of unadjusted and adjusted models of self-reported ownership and use of ITNs in **Kenya**. [1, 2]

	**Ownership of ITNs**		**Use of ITNs**	
	Unadjusted OR (95% CI)	Adjusted OR (95% CI)	Unadjusted OR (95% CI)	Adjusted OR (95% CI)
Total number of household members				
Small (<5)	Ref.	Ref.	Ref.	Ref.
Large (≥5)	0.81 (0.74-0.90)	**0.84***** (0.76-0.94)	0.61 (0.53-0.69)	**0.69***** (0.60-0.79)
Total number of children in the household				
1	Ref.		Ref.	
2 or more	1.04 (0.94-1.14)		1.04 (0.92-1.18)	
Type of place of residence				
Rural	Ref.	Ref.	Ref.	Ref.
Urban	1.40 (1.19-1.64)	0.96 (0.81-1.14)	2.21 (1.86-2.62)	**1.59***** (1.33-1.92)
Highest educational level				
Low	Ref.	Ref.	Ref.	Ref.
High	2.04 (1.81-2.31)	**1.75***** (1.50-2.03)	1.94 (1.67-2.26)	**1.40**** (1.18-1.66)
Main floor material				
Unfinished	Ref.	Ref.	Ref.	Ref.
Finished	1.61 (1.41-1.83)	0.89 (0.73-1.08)	1.89 (1.61-2.21)	1.03 (0.82-1.29)
Main wall material				
Unfinished	Ref.	Ref.	Ref.	Ref.
Finished	1.25 (1.11-1.41)	1.02 (0.84-1.23)	1.46 (1.25-1.71)	1.14 (0.93-1.40)
Main roof material				
Unfinished	Ref.		Ref.	Ref.
Finished	1.50 (0.98-2.30)		1.76 (1.02-3.01)	1.47 (0.67-3.22)
Wealth index				
Poor	Ref.	Ref.	Ref.	Ref.
Wealthy	1.83 (1.62-2.07)	**1.89***** (1.60-2.24)	1.83 (1.57-2.14)	1.13 (0.93-1.37)
Malaria parasite rate				
Low (<0.09)	Ref.	Ref.	Ref.	Ref.
High (≥0.09)	2.86 (2.47-3.31)	**2.75***** (2.33-3.24)	1.48 (1.24-1.78)	**1.55***** (1.29-1.88)
Average land travel friction per meter				
Low (<0.003)	Ref.	Ref.	Ref.	Ref.
High (≥0.003)	0.59 (0.50-0.70)	0.87 (0.74-1.04)	0.61 (0.52-0.73)	**0.84*** (0.70-1.00)

[1] Boldfaced numbers indicate statistical significance by confidence interval

[2] '*' symbol represents p-values (* p < 0.05, ** p < 0.01, *** p < 0.001)

Supplementary Table 7. Results of unadjusted and adjusted models of self-reported ownership and use of ITNs in **Malawi**. [1, 2]

	**Ownership of ITNs**		**Use of ITNs**	
	Unadjusted OR (95% CI)	Adjusted OR (95% CI)	Unadjusted OR (95% CI)	Adjusted OR (95% CI)
Total number of household members				
Small (<5)	Ref.	Ref.	Ref.	Ref.
Large (≥5)	**0.82***** (0.74-0.90)	**0.74***** (0.67-0.83)	**0.68***** (0.60-0.77)	**0.67***** (0.58-0.76)
Total number of children in the household				
1	Ref.	Ref.	Ref.	Ref.
2 or more	1.18 (1.07-1.29)	**1.34***** (1.22-1.48)	1.18 (1.05-1.33)	**1.38***** (1.22-1.57)
Type of place of residence				
Rural	Ref.	Ref.	Ref.	Ref.
Urban	1.47 (1.24-1.73)	0.93 (0.77-1.12)	1.83 (1.46-2.29)	**1.43**** (1.10-1.87)
Highest educational level				
Low	Ref.	Ref.	Ref.	Ref.
High	1.94 (1.73-2.18)	**1.61***** (1.43-1.82)	1.67 (1.45-1.93)	**1.43***** (1.23-1.67)
Main floor material				
Unfinished	Ref.	Ref.	Ref.	Ref.
Finished	1.73 (1.50-1.99)	**1.28***** (1.07-1.52)	1.47 (1.27-1.71)	1.23 (0.99-1.53)
Main wall material				
Unfinished	Ref.	Ref.	Ref.	
Finished	1.29 (1.15-1.45)	1.04 (0.92-1.17)	1.01 (0.88-01.17)	
Main roof material				
Unfinished	Ref.	Ref.	Ref.	Ref.
Finished	1.46 (1.32-1.61)	0.88 (0.77-1.00)	1.17 (1.02-1.33)	0.89 (0.74-1.07)
Wealth index				
Poor	Ref.	Ref.	Ref.	Ref.
Wealthy	1.75 (1.58-1.93)	**1.69***** (1.49-1.92)	1.18 (1.03-1.35)	1.07 (0.91-1.27)
Malaria parasite rate				
Low (<0.09)	Ref.	Ref.	Ref.	
High (≥0.09)	1.20 (1.06-1.36)	**1.32***** (1.16-1.51)	0.87 (0.73-1.04)	
Average land travel friction per meter				
Low (<0.003)	Ref.		Ref.	
High (≥0.003)	0.93 (0.81-1.06)		0.99 (0.75-1.17)	

[1] Boldfaced numbers indicate statistical significance by confidence interval

[2] '*' symbol represents p-values (* p < 0.05, ** p < 0.01, *** p < 0.001)

Supplementary Table 8. Results of unadjusted and adjusted models of self-reported ownership and use of ITNs in **Mozambique**. [1, 2]

	**Ownership of ITNs**		**Use of ITNs**	
	Unadjusted OR (95% CI)	Adjusted OR (95% CI)	Unadjusted OR (95% CI)	Adjusted OR (95% CI)
Total number of household members				
Small (<5)	Ref.	Ref.	Ref.	Ref.
Large (≥5)	1.27 (1.04-1.54)	1.17 (0.96-1.43)	0.69 (0.56-0.86)	**0.76*** (0.60-0.95)
Total number of children in the household				
1	Ref.		Ref.	
2 or more	0.89 (0.73-1.07)		1.10 (0.89-1.36)	
Type of place of residence				
Rural	Ref.	Ref.	Ref.	Ref.
Urban	1.56 (1.12-2.17)	0.83 (0.56-1.23)	1.32 (1.02-1.72)	**2.03***** (1.46-2.82)
Highest educational level				
Low	Ref.	Ref.	Ref.	Ref.
High	1.80 (1.36-2.39)	**1.43*** (1.06-1.91)	1.42 (1.09-1.86)	**1.45*** (1.08-1.94)
Main floor material				
Unfinished	Ref.	Ref.	Ref.	Ref.
Finished	2.51 (2.00-3.16)	**1.68**** (1.21-2.33)	0.67 (0.53-0.84)	**0.65**** (0.48-0.88)
Main wall material				
Unfinished	Ref.	Ref.	Ref.	Ref.
Finished	1.86 (1.46-2.37)	0.94 (0.68-1.29)	0.70 (0.56-0.88)	0.80 (0.59-1.07)
Main roof material				
Unfinished	Ref.	Ref.	Ref.	Ref.
Finished	2.32 (1.90-2.83)	**1.48*** (1.10-1.99)	0.69 (0.55-0.86)	0.85 (0.59-1.22)
Wealth index				
Poor	Ref.	Ref.	Ref.	Ref.
Wealthy	1.98 (1.56-2.52)	1.24 (0.90-1.70)	0.71 (0.57-0.90)	0.80 (0.59-1.09)
Malaria parasite rate				
Low (<0.09)	Ref.		Ref.	
High (≥0.09)	0.59 (0.26-1.33)		1.50 (0.95-2.38)	
Average land travel friction per meter				
Low (<0.003)	Ref.		Ref.	
High (≥0.003)	0.75 (0.55-1.01)		0.97 (0.72-1.32)	

[1] Boldfaced numbers indicate statistical significance by confidence interval

[2] '*' symbol represents p-values (* $p < 0.05$, ** $p < 0.01$, *** $p < 0.001$)

Supplementary Table 9. Results of unadjusted and adjusted models of self-reported ownership and use of ITNs in **Rwanda**. [1, 2]

	**Ownership of ITNs**		**Use of ITNs**	
	Unadjusted OR (95% CI)	Adjusted OR (95% CI)	Unadjusted OR (95% CI)	Adjusted OR (95% CI)
Total number of household members				
Small (<5)	Ref.	Ref.	Ref.	Ref.
Large (≥5)	1.29 (1.11-1.50)	1.08 (0.92-1.27)	0.79 (0.66-0.94)	**0.71***** (0.60-0.85)
Total number of children in the household				
1	Ref.		Ref.	Ref.
2 or more	1.00 (0.87-1.16)		1.30 (1.09-1.55)	**1.44***** (1.21-1.73)
Type of place of residence				
Rural	Ref.	Ref.	Ref.	Ref.
Urban	1.71 (1.29-2.27)	**0.71*** (0.52-0.97)	2.26 (1.66-3.07)	**1.80**** (1.25-2.59)
Highest educational level				
Low	Ref.	Ref.	Ref.	Ref.
High	2.20 (1.64-2.96)	1.13 (0.81-1.58)	2.22 (1.61-3.05)	**1.70**** (1.25-2.59)
Main floor material				
Unfinished	Ref.	Ref.	Ref.	Ref.
Finished	3.66 (2.80-4.79)	**1.51*** (1.04-2.18)	1.60 (1.30-1.97)	0.99 (0.75-1.32)
Main wall material				
Unfinished	Ref.	Ref.	Ref.	Ref.
Finished	2.97 (2.43-3.63)	**1.43**** (1.13-1.83)	1.46 (1.21-1.76)	1.06 (0.84-1.35)
Main roof material				
Unfinished	Ref.		Ref.	
Finished	1.16 (0.35-3.82)		0.95 (0.21-4.33)	
Wealth index				
Poor	Ref.	Ref.	Ref.	Ref.
Wealthy	4.00 (3.37-4.75)	**3.25***** (2.68-3.93)	1.43 (1.21-1.69)	**1.28**** (1.07-1.53)
Malaria parasite rate				
Low (<0.09)	Ref.		Ref.	
High (≥0.09)	1.03 (0.36-2.92)		0.57 (0.23-1.40)	
Average land travel friction per meter				
Low (<0.003)	Ref.		Ref.	
High (≥0.003)	0.79 (0.60-1.04)		0.90 (0.74-1.09)	

[1] Boldfaced numbers indicate statistical significance by confidence interval

[2] '*' symbol represents p-values (* p < 0.05, ** p < 0.01, *** p < 0.001)

Supplementary Table 10. Results of unadjusted and adjusted models of self-reported ownership and use of ITNs in **Tanzania**. [1, 2]

	**Ownership of ITNs**		**Use of ITNs**	
	Unadjusted OR (95% CI)	Adjusted OR (95% CI)	Unadjusted OR (95% CI)	Adjusted OR (95% CI)
Total number of household members				
Small (<5)	Ref.	Ref.	Ref.	Ref.
Large (≥5)	1.20 (1.05-1.37)	**1.21*** (1.04-1.42)	0.65 (0.54-0.79)	**0.75**** (0.62-0.90)
Total number of children in the household				
1	Ref.	Ref.	Ref.	
2 or more	1.15 (1.00-1.32)	**1.32***** (1.14-1.52)	1.12 (0.93-1.35)	
Type of place of residence				
Rural	Ref.	Ref.	Ref.	Ref.
Urban	2.69 (2.11-3.44)	**1.84***** (1.37-2.47)	3.13 (2.37-4.15)	**2.67***** (1.99-3.59)
Highest educational level				
Low	Ref.	Ref.	Ref.	Ref.
High	2.06 (1.58-2.68)	**1.33*** (1.00-1.77)	2.09 (1.56-2.80)	**1.38*** (1.03-1.86)
Main floor material				
Unfinished	Ref.	Ref.	Ref.	Ref.
Finished	2.51 (2.07-3.04)	**1.38*** (1.00-1.71)	1.91 (1.54-2.37)	1.23 (0.95-1.60)
Main wall material				
Unfinished	Ref.	Ref.	Ref.	
Finished	2.33 (1.86-2.91)	**1.98***** (1.55-2.54)	1.21 (0.97-1.52)	
Main roof material				
Unfinished	Ref.	Ref.	Ref.	Ref.
Finished	1.88 (1.53-2.31)	1.05 (0.83-1.32)	1.35 (1.09-1.67)	0.99 (0.78-1.27)
Wealth index				
Poor	Ref.	Ref.	Ref.	Ref.
Wealthy	2.24 (1.84-2.71)	1.24 (0.99-1.56)	1.50 (1.22-1.84)	0.85 (0.65-1.11)
Malaria parasite rate				
Low (<0.09)	Ref.	Ref.	Ref.	
High (≥0.09)	2.62 (1.85-3.71)	**4.15***** (2.94-5.87)	0.91 (0.65-1.27)	
Average land travel friction per meter				
Low (<0.003)	Ref.	Ref.	Ref.	
High (≥0.003)	0.64 (0.49-0.85)	**0.72*** (0.54-0.96)	0.80 (0.61-1.05)	

[1] Boldfaced numbers indicate statistical significance by confidence interval

[2] '*' symbol represents p-values (* $p < 0.05$, ** $p < 0.01$, *** $p < 0.001$)

Supplementary Table 11. Results of unadjusted and adjusted models of self-reported ownership and use of ITNs in **Uganda**. [1, 2]

	Ownership of ITNs		Use of ITNs	
	Unadjusted OR (95% CI)	Adjusted OR (95% CI)	Unadjusted OR (95% CI)	Adjusted OR (95% CI)
Total number of household members				
Small (<5)	Ref.	Ref.	Ref.	Ref.
Large (≥5)	0.80 (0.71-0.90)	0.89 (0.79-1.00)	0.59 (0.52-0.67)	**0.58***** (0.50-0.67)
Total number of children in the household				
1	Ref.		Ref.	Ref.
2 or more	1.01 (0.91-1.14)		1.27 (1.11-1.44)	**1.59***** (1.38-1.83)
Type of place of residence				
Rural	Ref.	Ref.	Ref.	Ref.
Urban	1.40 (1.15-1.72)	0.88 (0.70-1.12)	1.64 (1.33-2.01)	**1.33*** (1.06-1.67)
Highest educational level				
Low	Ref.	Ref.	Ref.	Ref.
High	1.94 (1.73-2.18)	**1.61***** (1.43-1.82)	2.24 (1.91-2.63)	**2.00***** (1.68-2.38)
Main floor material				
Unfinished	Ref.	Ref.	Ref.	Ref.
Finished	1.84 (1.58-2.16)	1.18 (0.94-1.48)	1.43 (1.24-1.66)	**1.27*** (1.04-1.56)
Main wall material				
Unfinished	Ref.	Ref.	Ref.	Ref.
Finished	1.63 1.42-1.87)	1.02 (0.84-1.25)	1.17 (1.01-1.35)	1.03 (0.85-1.25)
Main roof material				
Unfinished	Ref.	Ref.	Ref.	Ref.
Finished	1.27 (1.08-1.49)	**0.68***** (0.56-0.82)	0.71 (0.61-0.83)	**0.54***** (0.46-0.65)
Wealth index				
Poor	Ref.	Ref.	Ref.	
Wealthy	1.98 (1.73-2.28)	**1.97***** (1.64-2.36)	1.04 (0.91-1.19)	
Malaria parasite rate				
Low (<0.09)	Ref.	Ref.	Ref.	
High (≥0.09)	0.70 (0.54-0.91)	0.85 (0.66-1.09)	1.11 (0.89-1.40)	
Average land travel friction per meter				
Low (<0.003)	Ref.		Ref.	Ref.
High (≥0.003)	0.85 (0.71-1.00)		0.74 (0.62-0.90)	0.90 (0.74-1.09)

[1] Boldfaced numbers indicate statistical significance by confidence interval

[2] '*' symbol represents p-values (* p < 0.05, ** p < 0.01, *** p < 0.001)

Supplementary Table 12. Results of unadjusted and adjusted models of self-reported ownership and use of ITNs in **Zambia**. [1, 2]

	Ownership of ITNs		Use of ITNs	
	Unadjusted OR (95% CI)	Adjusted OR (95% CI)	Unadjusted OR (95% CI)	Adjusted OR (95% CI)
Total number of household members				
Small (<5)	Ref.	Ref.	Ref.	Ref.
Large (≥5)	1.14 (1.01-1.29)	1.10 (0.97-1.25)	0.68 (0.60-0.77)	**0.65******** 0.57-0.74)
Total number of children in the household				
1	Ref.		Ref.	Ref.
2 or more	1.02 (0.91-1.15)		1.17 (9.03-1.32)	**1.23**** (1.08-1.40)
Type of place of residence				
Rural	Ref.	Ref.	Ref.	Ref.
Urban	0.75 (0.64-0.88)	**0.48***** (0.38-0.60)	0.81 (0.70-0.94)	1.21 (0.99-1.47)
Highest educational level				
Low	Ref.	Ref.	Ref.	
High	1.29 (1.15-1.45)	**1.46***** (1.29-1.65)	1.10 (0.97-1.24)	
Main floor material				
Unfinished	Ref.		Ref.	Ref.
Finished	1.04 (0.89-1.21)		0.69 (0.60-.79)	0.85 (0.70-1.04)
Main wall material				
Unfinished	Ref.	Ref.	Ref.	Ref.
Finished	0.78 (0.68-0.90)	**0.68***** (0.58-0.79)	0.78 (0.67-0.89)	1.04 (0.88-1.22)
Main roof material				
Unfinished	Ref.		Ref.	Ref.
Finished	1.04 (0.90-1.20)		0.66 (0.57-0.75)	**0.77*** (0.63-0.95)
Wealth index				
Poor	Ref.	Ref.	Ref.	Ref.
Wealthy	1.23 (1.07-1.42)	**2.08***** (1.77-2.45)	0.67 (0.59-0.76)	0.89 (0.74-1.06)
Malaria parasite rate				
Low (<0.09)	Ref.		Ref.	Ref.
High (≥0.09)	0.95 (0.79-1.14)		1.28 (1.10-1.50)	1.10 (0.92-1.32)
Average land travel friction per meter				
Low (<0.003)	Ref.	Ref.	Ref.	Ref.
High (≥0.003)	1.23 (1.01-1.48)	1.10 (0.86-1.41)	1.23 (1.05-1.45)	1.06 (0.88-1.29)

[1] Boldfaced numbers indicate statistical significance by confidence interval

[2] '*' symbol represents p-values (* $p < 0.05$, ** $p < 0.01$, *** $p < 0.001$)

Supplementary Table 13. Results of unadjusted and adjusted models of self-reported ownership and use of ITNs in **Zimbabwe**. [1, 2]

	**Ownership of ITNs**		**Use of ITNs**	
	Unadjusted OR (95% CI)	Adjusted OR (95% CI)	Unadjusted OR (95% CI)	Adjusted OR (95% CI)
Total number of household members				
Small (<5)	Ref.	Ref.	Ref.	
Large (≥5)	1.21(1.06-1.39)	**1.22*** (1.07-1.40)	0.83 (0.67-1.01)	
Total number of children in the household				
1	Ref.		Ref.	
2 or more	0.99 (0.87-1.13)		1.17 (0.99-1.39)	
Type of place of residence				
Rural	Ref.		Ref.	Ref.
Urban	0.84 (0.67-1.05)		1.38 (1.02-1.86)	**1.38*** (1.02-1.86)
Highest educational level				
Low	Ref.		Ref.	
High	1.10 (0.95-1.28)		1.12 (0.90-1.39)	
Main floor material				
Unfinished	Ref.	Ref.	Ref.	
Finished	1.24 (1.03-1.49)	0.97 (0.78-1.22)	0.92 (0.72-1.17)	
Main wall material				
Unfinished	Ref.	Ref.	Ref.	
Finished	1.32 (1.06-1.64)	1.17 (0.93-1.48)	0.91 (0.69-1.21)	
Main roof material				
Unfinished	Ref.		Ref.	
Finished	1.16 (0.98-1.38)		0.96 (0.76-1.20)	
Wealth index				
Poor	Ref.	Ref.	Ref.	
Wealthy	1.33 (1.11-1.58)	**1.28*** (1.03-1.60)	0.99 (0.77-1.28)	
Malaria parasite rate				
Low (<0.09)	Ref.		Ref.	
High (≥0.09)	0.99 (0.46-2.11)		1.51 (0.76-3.00)	
Average land travel friction per meter				
Low (<0.003)	Ref.		Ref.	
High (≥0.003)	0.97 (0.71-1.33)		0.85 (0.56-1.28)	

[1] Boldfaced numbers indicate statistical significance by confidence interval

[2] '*' symbol represents p-values (* p < 0.05, ** p < 0.01, *** p < 0.001)

References

1. ICF: **Demographic and Health Surveys Standard Recode Manual for DHS7**. In. Rockville, Maryland, U.S.A.:ICF: The Demographic and Health Surveys Program; 2018.
2. Florey L, Taylor C: **Using household survey data to explore the effects of improved housing conditions on malaria infection in children in Sub-Saharan Africa**. In: *DHS Analytical Studies No 61.* Rockville, Maryland, USA: ICF International; 2016.
3. Rutstein SO, Johnson K: **The DHS wealth index**. In: *DHS Comparative Reports No 6.* Calverton, Maryland, USA: ORC Macro; 2004.
4. Hay SI, Guerra CA, Gething PW, Patil AP, Tatem AJ, Noor AM, Kabaria CW, Manh BH, Elyazar IRF, Brooker S *et al*: **A world malaria map: plasmodium falciparum endemicity in 2007**. *PLoS Medicine* 2009, **6**(3):0286-0302.
5. Hamilton M, Mahiane G, Werst E, Sanders R, Briët O, Smith T, Cibulskis R, Cameron E, Bhatt S, Weiss DJ: **Spectrum-malaria: A user-friendly projection tool for health impact assessment and strategic planning by malaria control programmes in sub-Saharan Africa**. *Malaria journal* 2017, **16**(1):68.
6. Hay SI, Snow RW: **The Malaria Atlas Project: developing global maps of malaria risk**. *PLoS medicine* 2006, **3**(12):e473.
7. Moyes CL, Temperley WH, Henry AJ, Burgert CR, Hay SI: **Providing open access data online to advance malaria research and control**. *Malaria Journal* 2013, **12**(1):161.
8. Weiss DJ, Nelson A, Gibson HS, Temperley W, Peedell S, Lieber A, Hancher M, Poyart E, Belchior S, Fullman N *et al*: **A global map of travel time to cities to assess inequalities in accessibility in 2015**. *Nature* 2018, **553**(7688):333-336.
9. Friedl MA, Sulla-Menashe D, Tan B, Schneider A, Ramankutty N, Sibley A, Huang X: **MODIS Collection 5 global land cover: Algorithm refinements and characterization of new datasets**. *Remote sensing of Environment* 2010, **114**(1):168-182.
10. Linard C, Gilbert M, Snow RW, Noor AM, Tatem AJ: **Population distribution, settlement patterns and accessibility across Africa in 2010**. *PloS one* 2012, **7**(2):e31743.
11. **WorldPop Project** [www.worldpop.org]
12. Africa ECf: **The Demographic Profile of African Countries**. In.: United Nations; 2016.
